# Structural Analysis of Prolyl Oligopeptidases Using Molecular Docking and Dynamics: Insights into Conformational Changes and Ligand Binding

**DOI:** 10.1371/journal.pone.0026251

**Published:** 2011-11-23

**Authors:** Swati Kaushik, Ramanathan Sowdhamini

**Affiliations:** National Centre for Biological Sciences, Tata Institute of Fundamental Research, Bangalore, India; University of South Florida College of Medicine, United States of America

## Abstract

Prolyl oligopeptidase (POP) is considered as an important pharmaceutical target for the treatment of numerous diseases. Despite enormous studies on various aspects of POPs structure and function still some of the questions are intriguing like conformational dynamics of the protein and interplay between ligand entry/egress. Here, we have used molecular modeling and docking based approaches to unravel questions like differences in ligand binding affinities in three POP species (porcine, human and *A. thaliana*). Despite high sequence and structural similarity, they possess different affinities for the ligands. Interestingly, human POP was found to be more specific, selective and incapable of binding to a few planar ligands which showed extrapolation of porcine POP in human context is more complicated. Possible routes for substrate entry and product egress were also investigated by detailed analyses of molecular dynamics (MD) simulations for the three proteins. Trajectory analysis of bound and unbound forms of three species showed differences in conformational dynamics, especially variations in β-propeller pore size, which was found to be hidden by five lysine residues present on blades one and seven. During simulation, β-propeller pore size was increased by ∼2 Å in porcine ligand-bound form which might act as a passage for smaller product movement as free energy barrier was reduced, while there were no significant changes in human and *A. thaliana* POPs. We also suggest that these differences in pore size could lead to fundamental differences in mode of product egress among three species. This analysis also showed some functionally important residues which can be used further for *in vitro* mutagenesis and inhibitor design. This study can help us in better understanding of the etiology of POPs in several neurodegenerative diseases.

## Introduction

Serine proteases are the examples where relationship between homology and substrate specificity is still a paradox. Despite high sequence identity in any two proteases, they can be quite specific towards a given macromolecular substrate [1]. Unlike other traditional serine proteases like trypsin and subtilisins, POP cleaves peptides which are smaller than 30 amino acids in length [2]–[4]. Binding of small peptides to POP is essential for many physiological processes and has gained insights as a target for the treatment of numerous disorders like depression, amnesia, schizophrenia, trypanosomiasis, bipolar affective disorder etc [5]–[7]. A recent study also showed lower plasma POP activity in patients of multiple sclerosis [8]. This peptidase has been implicated in neurodegeneration, as well as in the modulation of the inflammatory response [8]. In spite of enormous studies of role of POP in various diseases the precise biological function of protein is still unknown.

POP is a widely distributed enzyme and has been cloned and isolated from several sources [9]–[15]. The X-ray crystal structure of enzyme shows unique domain architecture with a catalytic α/β hydrolase domain and an unusual β-propeller domain. Propeller domain is based on radially arranged seven-fold repeat of four-stranded antiparallel β sheets. In the case of POPs, this domain is considered to be of the “open-velcro” topology, where first and seventh blades are connected only through hydrophobic interactions. The catalytic triad (Ser 554, His 680, and Asp 641) is hidden and located at the interface of two domains. This unique propeller which is absent in other α/β hydrolases, acts as a lid to hide the active site and also as a selectivity or gating filter, thereby allowing only small peptides to reach active site [16]; despite central inter-domain cavity, that can accommodate bigger ligands. Various experimental studies have suggested concerted movement of propeller and peptidase domains are necessary for enzyme activity [17].

Evolutionary studies of POP family shows that plant POP diverge before mammalian POP [18]. Phylogenetic analysis showed that POP is the most conserved enzyme in POP family [18]. In animals, POP is widely distributed with high concentration found in the brain, and its involvement in the control of several mammalian peptide hormones signaling pathways have been studied extensively [19]–[20]. As abnormal POP activity is found to be linked with various neurological disorders, for preclinical trials porcine POP is widely studied as model to identify potential, potent and selective inhibitors [26]. Most of the reported inhibitors entered clinical trials [27], but their use as drug has not been reported yet. Unlike other members of POP family crystal structure of the POP does not explain the possible passage for substrate/product entry/egress therefore substantial conformational changes are expected. Despite the enormous amount of data from both experimental and computational studies, mechanisms of substrate/inhibitor entry and product egress are still unknown [28], [29]. Recent crystal structure of bacterial POP (*S. capsulata*) in open form suggests large opening between two domains for substrate/inhibitor entry, while presence of inhibitor shuts the opening [30]. Comparative analysis of the two structures highlights role of inter-domain dynamics. However, from other crystal structures of closed form of mammalian POPs, porcine POP for instance, have various hydrogen bonds that act like a bridge in connecting two domains along with numerous loops from β-propeller domain which behaves like a covering sheath. Sequence analysis of these two POPs suggests salt bridges, present in bacterial POPs that function as a latch for inter-domain opening movement, is not conserved in porcine [27]. This highlights the fact that bacterial POP may not represent a common and unified mechanism for action of every POP enzyme of other species. So, it is also anticipated that different POP species can have different substrate entry or product egress mechanisms [27]. Previous studies have shown that plant POPs are distant members of same family but till now their function in plants is not known. Unavailability of drugs and no successful clinical trials on human has inspired us to carry out this analysis to better understand the differences of POP among different species if any.

**Figure 1:**
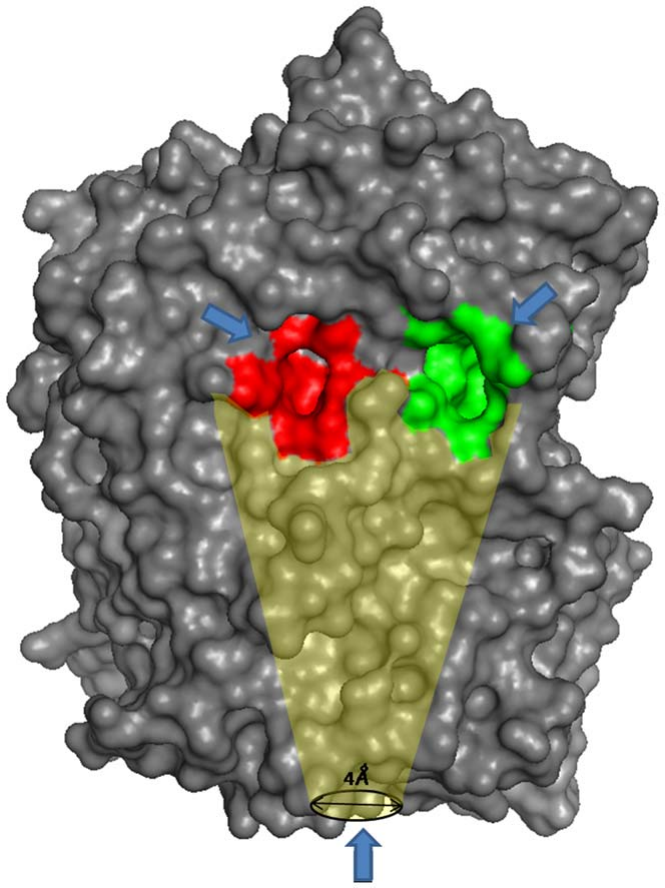
Cavities present in POP. Bottom arrow indicates β-propeller cavity which continues till active site and form another smaller cavity near active site shown in red color (Tarrago et al 2009), inter-domain cavity is shown in green color.

In the present study, we have carried out in-depth analysis and comparison of POPs from three different species human, porcine and plant (*A. thaliana*) in terms of ligand specificity and binding. This comparison was done to better understand the differences and conformational dynamics of the protein. We have focused on two main issues firstly, to what extant extrapolation of porcine POP to human POP is correct and secondly investigating the possible passage for substrate/product entry/egress. To unravel the above mentioned questions we have applied computational based approaches like molecular docking and dynamics. We were interested in studying plant POPs as they are distant members of same family so it would be interesting to see from evolutionary perspectives like how a distant member of same family behaves as compared to newly diverged members. Moreover, few naturally occurring plant molecules are reported as POP inhibitors having therapeutic applications but nothing much is known about their function and presence in plant system [21]–[25]. Three possibilities of substrate entry were considered: (1) Movement of side chains present at β-propeller pore will allow annulus of tunnel to become broad (2) the first and seventh blade will move apart, thereby increasing pore size (3) Hinge-like motion between two domains causing separation, thereby substrate entry [31]. Further comparison of all the reported cavities (Figure 1) present in protein, that includes β-propeller, inter-domain (between two domains reported by Polgar and coworkers, [28]) and a recently reported cavity (by Tarrago and coworkers found using cryo-electron microscopy which is present just above active site [32]) was carried out for unrevealing the possible entry/exit mechanism.

Exhaustive examination and analysis of these POP species showed differences in binding affinities of different inhibitors which include differences in binding in porcine and human POPs. Interestingly, human POP was found to be more selective and specific and also showed hindrance in binding to some ligands which binds well to porcine and *A. thaliana*, this indicates the extrapolation of porcine to human POP will be difficult in all cases. Detailed analyses of molecular dynamics trajectories of bound/unbound form reveals the dynamic conformational changes associated with the ligand binding in three POP species. Striking differences in β-propeller pore size in bound forms of three POP species were noticed. We have also shown that smaller product movement is possible from β-propeller in porcine, while it may differ in other two species.

## Methods

### Alignment and model preparation

Protein sequence of *A. thaliana* POP was retrieved from TIGR (The Institute of Genomic Research) database [33]. The sequence obtained was subjected to BLAST (Basic Local Alignment Tool) against PDB (Protein Data Bank) to extract information about suitable structural template and to PSIPRED (Protein Structure Prediction Server) for predicting secondary structural elements, respectively 34–36. Sequence alignment was done using CLUSTALW [37] and Joy4.0 program was used to annotate the alignment using three dimensional structural information of template [38]. DSSP (Database of secondary structure assignment) was employed for the assignment of secondary structure [39]. Crystal structure of porcine POP (PDB ID: 1E5T) was used as a template for the construction of model. Alignment of query and template was considered to build the model using MODELLER (version 9.1, [40]). A set of 100 models were generated, from which lower energy structure according to DOPE (Discrete optimized protein energy) score was used for further processes. Geometric inaccuracies of the structural model were evaluated by subjecting the model to PDB-ADIT validation server, which validates using PROCHECK [41]–[42]. The structure was further energy minimized with the SYBYL software package (version 7.1) using Tripos forcefield [43]. For first 500 steps minimization was carried out with steepest descent which was followed by 200 iterations of conjugate gradient with distant dependent dielectric constant equal to 1, non-bonded interaction cutoff value of 8 and was terminated at the convergence of 0.05 kcal mol Å^−1^. The final structure was validated again using the PROCHECK and PROSA that checks for high energy regions of the modeled structure [44]. Structural model was structurally aligned with the experimental structure and rendered using PyMOL.

### Ligand selection

The set of ligand molecules studied in this work include known inhibitor Z pro prolinal (ZPR) and related molecules like Z-prolyl pyrrolidine, Z-prolyl prolinol, Z-prolyl azetidine, Z-phenyl alanine azetidine, pyrazinone [19], [45]. Besides this, naturally occurring plant flavonoid inhibitors baicalein [24] and berberine [25] were also used which are known porcine POP inhibitors. These ligands were either downloaded from PubChem or were constructed using CHEMDRAW software. Few other ligands like Y-29794 [46]–[48], UAMC [49], ono-1603 [50]–[51], S-17092 [52]–[56], SUAM-1221 [57]–[61], and JTP4819 [62]–[67] were also considered for this study. Coordinates were saved and subjected to CORINA for two dimensional to three dimensional conversions. The three dimensional structure of porcine POP (1QFS), which is ligand bound form with Z pro prolinal was downloaded from PDB. This structure was determined using X ray crystallography with resolution of 2 Å. The energy of ligand molecules were minimized for 200 runs using steepest descent followed by 100 runs of conjugate gradient using SYBYL software. Each of the minimization methods were carried out with Tripos forcefield.

### Docking studies

In order to carry out the docking simulation, AutoDock 4.0 [68] was used. It is one of the most suitable methods for performing molecular docking of ligand to their macromolecular receptors. Here rigid docking protocol was considered, where both ligands and receptors were kept rigid. The Graphical User Interface program “Autodock tools” was used to prepare, run and analyze the docking simulations. Polar hydrogen's were added into the receptor PDB file for the preparation of protein in docking simulation. Gasteiger charge was assigned and non-polar hydrogens were merged to the ligands. Autodock requires grid maps, and grid must surround the region of interest in the macromolecule. In this study one of the catalytic triad residues Ser 554 was selected as the active site residue. So, the grid was centered on this catalytic active region of the receptor. The grid box size was set at 60, 60 and 60 Å (x, y and z). For blind docking grid size was increased so that entire protein can be accommodated inside grid where grid dimensions were 68, 62 and 82 Å (x, y and z). AutoGrid program was used to produce grid maps. The spacing between grid points was 0.37 Å and 1 Å for active site and blind docking respectively. The Lamarckian Genetic Algorithm (LGA) was used to search for the best conformers. During the docking process, a maximum of 100 conformers were considered for each compound. The population size was set to 150 and the individuals were initialized randomly. Maximum number of energy evaluation was set to 2.5×10^−6^, maximum number of generations to 27000, maximum number of top individual that automatically survived to 1, with a mutation rate of 0.02 and a crossover rate of 0.80.

### Electrostatic charge distribution

The Adaptive Poisson-Boltzmann Solver (APBS) program was employed to calculate the electrostatic charge distribution of porcine and human crystal structure [70]. Electrostatic charge distribution was also mapped on the theoretical structure of *A. thaliana* and comparison was made with respect to possible cavities present in the protein. PyMOL was used for the visualization of the surface representation.

### Molecular Dynamics Calculations

MD calculations at room temperature were carried out using Gromacs [71] software with gromos96 force field for porcine (1QFS), human (3DDU) [72] and the model of *A. thaliana* POP (generated in this work). Proteins were immersed in cubic water box of spce water model with box edges 9 Å from molecule periphery. 21 sodium ions were added to achieve electroneutrality. The system was subjected to pre-equilibration process that involves minimization using steepest descent for 6000 steps followed by position restrained dynamics for 20 ps. MD calculations were performed at constant volume and temperature for both bound and unbound forms each for 20 ns of three POP species. The system was simulated under periodic boundary conditions with cutoffs of 10 Å each for electrostatic and Van der Waals terms. Replicate of the MD runs were also performed using different seed numbers. Snapshots were collected after every 1 ps. Prodrug server was used to obtain drug parameters [73]. Strong/short (distance between nucleophiles <2.8 Å) hydrogen bonds between two domains were calculated using HBPLUS [69] for the comparison among species.

## Results

### Alignment and model preparation

**Figure 2:**
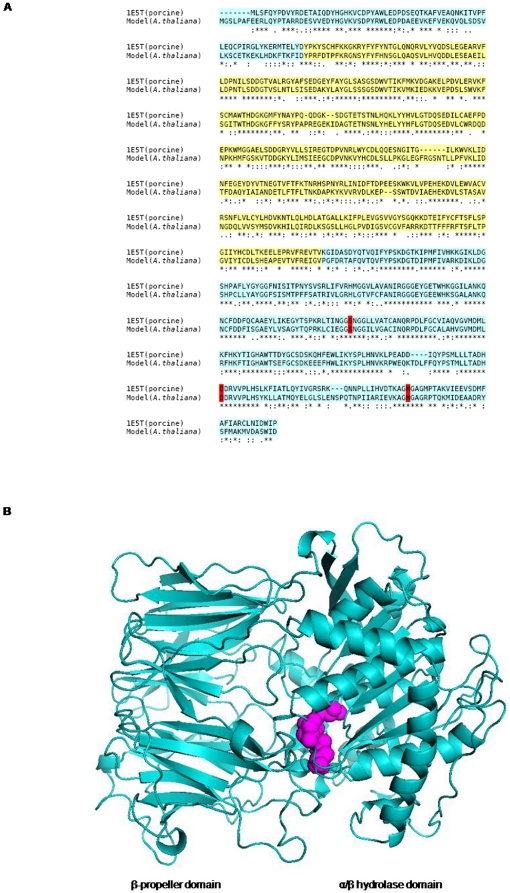
Model generation. **a**) alignment between query (At1g20380) and template (PDB ID: 1E5T, porcine) generated by CLUSTALW, sequence identity was found to be 53%. α/β hydrolase domain is cyan colored and β-propeller is shown in yellow color. Active site residue conservation is shown in red color **b**) homology model of *A. thaliana* POP generated using porcine POP as a template. Catalytic triad is shown in magenta color.

For generating structural model of *A. thaliana* POP BLAST search was performed, using plant POP as a query against PDB database, suggests POP from porcine brain (PDB ID: 1E5T, resolution 1.7 Å) as a potential template for modeling of plant POP. Plant and porcine POPs are definite homologues with sequence identity of 53% (Figure 2a). Alignment was analyzed to check preservation of conserved residues along with catalytic triad residues present towards C-terminal of the sequence. There was good agreement between the secondary structural elements of both sequences. Catalytic triad was also found to be in similar orientations in theoretical structure as in porcine POP (Figure 2b). Overall, 95% of residues were present in allowed region representing a good model quality.

### Electrostatic charge distribution

**Figure 3:**
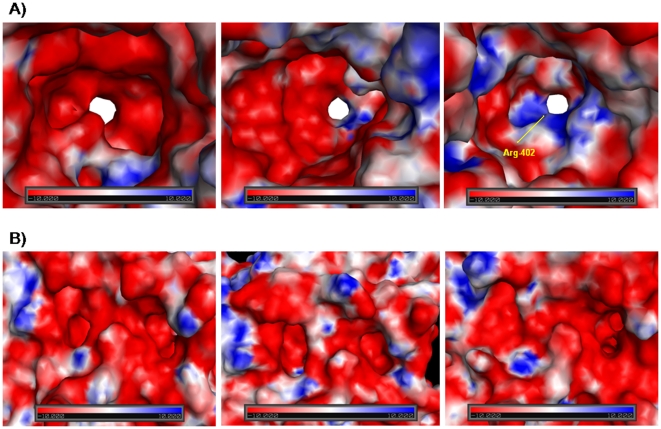
Electrostatic charge distribution of POP of three species. **a**) down the β-propeller pore view of electrostatic potential. Three species showed differences in electrostatics. *A. thaliana* POP showed more positive potential; similarly differences were also present in human and porcine POP proteins **b**) Electrostatic potential of inter-domain cavity and recently reported cavity (Tarrango et al 2009). In *A. thaliana* later cavity was hidden.

Electrostatic charge distribution was mapped on all three POP species for finding species specific surface differences. Analysis of electrostatic charge distribution reveals deviations in surface electrostatics at different cavities present in POP proteins of three species (Figure 3). Comparison was done with respect to known cavities present in porcine protein of known structure to study differences in possible initial binding sites of ligand. In order to check whether there are species-specific differences, we have compared electrostatic distribution on the surfaces of human and *A. thaliana* POP with porcine POP. Intensity of positive potential was found to be varying in the three protein structures. Striking differences were observed among three species: mouth of the β-propeller cavity was found to have more positive potential in human POP as compared to porcine, while *A. thaliana* POP shows even higher positive potential than other two because of the presence of an extra Arg402 (Figure 3a, Figure S1). These differences were found to be concentrated in loop regions of first and seventh blades of β-propeller. Blade 1 of both porcine and human POP was having high positive surface potential. Further, the comparison of cavity present just above the active site by Tarrago and coworkers [32] suggests that surrounding residues have higher positive potential in humans, as compared to porcine, while it was found to be near-neutral in *A. thaliana* (Figure 3b). These differences in the protein electrostatics may lead to difference in the recognition and initial binding of the ligands in three species.

### Molecular Docking

Most of the POP inhibitors contain a proline skeleton (exactly a proline group or proline analogs). The first effective inhibitor which was discovered was Z-pro prolinal. The set of ligand molecules studied in this work include Z-pro prolinal, its related compounds and various other molecules which were tested for preclinical trials (Y-29794, UAMC, ono-1603, S-17092, SUAM-1221, and JTP4819) in other organisms like rat, porcine etc. Molecular docking simulations were conducted using AutoDock4. For all the docking runs, minimum energy conformation was selected.

### Inhibitor docking

**Figure 4:**
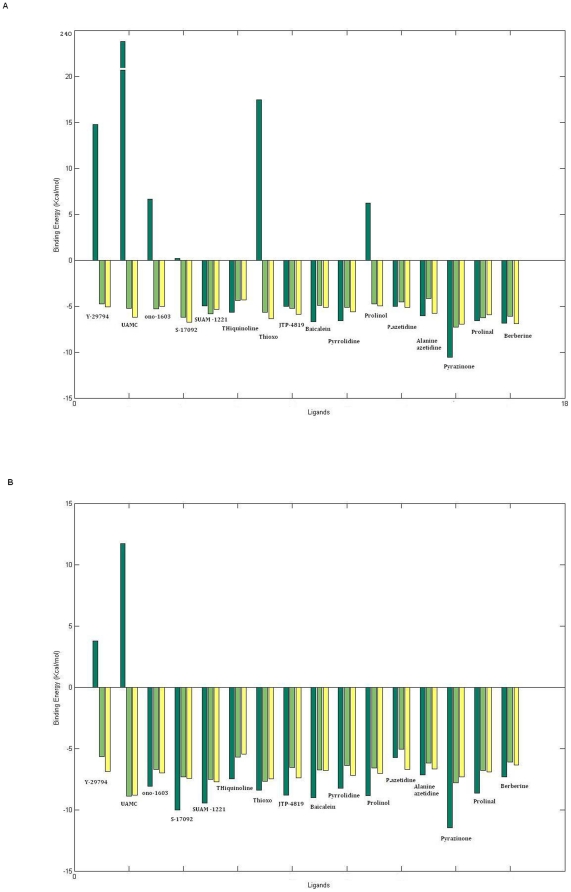
Rigid and flexible docking binding scores. **a**) Rigid docking scores of human, porcine and *A. thaliana* POP. Some of the ligands showed hindrance in binding to human POP as revealed by their positive energy scores. Different ligands showed difference in binding affinities to three species of POP. **b**) Flexible docking scores of human, porcine and *A. thaliana* POP. After inducing flexibility in ligands still two of the ligands were incapable of binding to human POP.

In order to identify residues potentially involved in ligand binding and to analyze its functional importance, the receptor was docked to different ligands. Initially, rigid docking approach was used. Each docking pose was analyzed and compared in three species. Ligands were found to be docked in different conformations and with varying affinities, as reflected by docking scores (Figure 4). Interestingly, few of the ligands Y-29794, UAMC, ono-1603, S-17092, thioxo and Z-pro prolinol showed some hindrances in binding to human POP as revealed by their positive binding energies (Figure 4a).

For ligands that showed no evidence of binding (positive energies) using rigid docking approach, docking runs were repeated after subjecting the ligands to flexible docking, where flexibility was induced in ligands alone. This was done in order to provide more optimal interactions between protein and ligand during the docking. Ligands were again found to bind with different affinities in three POPs. Some of the ligands that were showing poor scores in rigid docking were found to bind well when the ligand conformation was permitted to be flexible. However, still two planar ligands Y-29794, UAMC were incapable of binding. This binding incapability suggests difference in selectivity/affinity of ligands for binding to human POP (Figure 4b). Their binding orientation was found to be different than porcine and *A. thaliana* POP. In Y-29794, change in orientation of binding leads to interaction with more polar residues like Thr481, Ser479, Tyr471and Asn483 which causes hindrance in binding while in porcine smaller residues like Ala and Gly are present. Similarly, UAMC binds in different orientation causing short contact in human POP. Plant based naturally occurring animal POP inhibitors baicalein and berberine were found to bind with high affinities to *A. thaliana* POP protein too, which shows they are effective ligands but their function in plants is still not known. Other ligands were also found to bind well to *A. thaliana* POP, but whether their function (inhibitor) is similar in plants is still an area to be explored.

In order to identify possible regions of protein where ligand (Z-pro-prolinal) can bind effectively, blind docking approach was used. Grid was increased to dimensions which can accommodate entire protein inside and then comparisons among three POPs were carried out. In porcine POP, by following the various docking poses, it appears that the ligand can make specific path right from mouth of β-propeller to active site of protein, while it was not very well distributed and found to be present at specific places like end of α/β hydrolase domain and in middle of β-propeller cavity in human (Figure S2). In order to validate whether binding is not random, dummy molecule sucrose was also docked to porcine POP (Figure S3) as a negative control. Sucrose was found to bind all over protein (unspecific binding) while ligand ZPR had more specific binding. In human POP, ligand binding was restricted to some particular regions only, though CASTp [74] results showed β-propeller cavity is more voluminous in human (11323 Å^3^) as compared to porcine (10471 Å^3^). Besides this, binding of Z-pro-prolinal was more feasible in human (binding energy −10.1 to 3.1 kcal/mol), as compared to porcine (binding energy −7.1 to −4.3 kcal/mol) and *A. thaliana* (binding energy −5.2 to −3.3 kcal/mol) POPs.

### Substrate/Product docking

All short peptides with an internal proline residue are potential POP substrate. Blind docking of POP with substrate angiotensin IV (V-Y-I-H-**P**-F) was also carried out to known possible regions of binding [75]. This was found to be similar (data not shown) as when inhibitor Z-pro-prolinal was docked. Binding was restricted to some particular regions in human, unlike porcine POP, but binding feasibility was higher (energy/residue −3.3 kcal/mol in human, −1.9 kcal/mol in porcine). Similarly, N terminal (V-Y-I-H-**P**) and C terminal (F) products were docked to human and porcine POP. C-terminal product was docked successively after docking N-terminal product. Comparison of percentage docked poses of substrate and product showed some differences with respect to known cavities and domains (Figure S4). It was found that substrate has tendency to bind at particular regions like β-propeller cavity, functionally important loop (residues 192–205) and towards α/β hydrolase domain of protein while products were present at multiple sites which were reported till now including inter-domain cavities reported by Polgar and coworkers [28] and Tarrago and coworkers [32], reflecting its multiple egress sites.

### Molecular dynamics

Overall, nine simulations were run, with a total duration of ∼0.21 µs. However, rather than running a single long simulation, we explored the effect of presence/absence of ligand (ZPR, each 20 ns). The rationale behind these multiple simulations was to understand differences in dynamics of POP protein in three species, also in the ligand unbound and bound conformations.

Briefly simulations performed with replicates were:

1. Six simulations each 20 ns of bound/unbound form of human, porcine and *A. thaliana*.
2. One simulation (20 ns) for unbound form of human when lysines were mutated.
3. One 45 ns simulation where ligand was at mouth of β-propeller pore in porcine POP.

#### Simulation with bound ZPR/unbound forms

**Figure 5:**
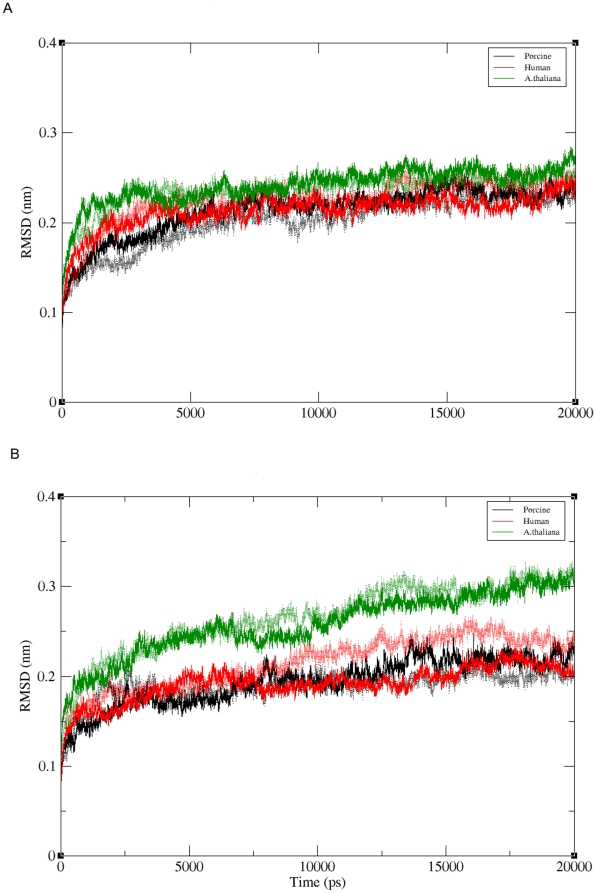
Root mean square deviations (rmsd) of backbone Cα of three POP species during simulation. **a**) rmsd of bound form of porcine (black), human (red) and *A. thaliana* (green) POPs **b**) rmsd of unbound form of porcine, human and *A. thaliana* POPs. Dotted line indicates rmsd of replicate runs.

Conformational stability was assessed by the drift of the protein from the crystal structure as measured by root-mean-square deviation (rmsd) of C^α^ atoms from their initial coordinates (Table 1). During simulation, all the three proteins were found to be stable (Figure 5). Unbound form of *A. thaliana* showed higher deviations as compared to porcine and human POP, overall β-propeller domain showed higher fluctuations than α/β hydrolase domain (Figure S5). In the ligand-bound form, rmsd of the drug position showed more variability and fluctuations in binding pocket of porcine POP hence more mobility of drug as compared to human and *A. thaliana* (Figure S6). R_g_ plots showed a slight decrease (∼0.3 Å) in bound forms of POPs. Flexibility of residues was seen by measuring rmsd during simulation. Like other studies done before (Polgar and coworkers [28] and Tarrago and coworkers [32]), we also found that loops present at inter-domain region showed high fluctuations; besides, residues 266–275 of β-propeller blade 4 also showed very high fluctuation, along with other loops connecting β-strands of blade 1, 2, 5 and 7. This, together with the higher positive potential at the propeller mouth of *A. thaliana* POP, might suggest that the substrate entry is more likely by inter-domain movements.

**Table 1:** Root mean square deviations (rmsd) of simulations carried out. rmsd of replicate run is shown in brackets.

Simulation	Duration[ns]	Form	C^á^ rmsd Å[D1 +D2]	*D1 [β-propeller] [Å]	*D2 [α/β hydrolase] [Å]
**Porcine**	20	Bound	1.77 (1.64)	1.80 (1.49)	1.59 (1.31)
	20	Unbound	2.02 (1.63)	2.04 (1.53)	1.42 (1.13)
***A. thaliana***	20	Bound	2.06 (1.74)	2.00 (1.72)	1.79 (1.44)
	20	Unbound	2.20 (2.13)	1.77 (1.69)	2.15 (1.94)
**Human**	20	Bound	2.02 (1.96)	1.89 (1.83)	1.82 (1.25)
	20	Unbound	1.64 (1.95)	1.63 (1.97)	1.51 (1.55)
**Porcine**	45	Drug at β propeller	1.45 (1.72)	1.40 (1.66)	1.32 (1.63)

*Deviations of two domains were also calculated separately.

#### Conformational changes during simulation

**Figure 6:**
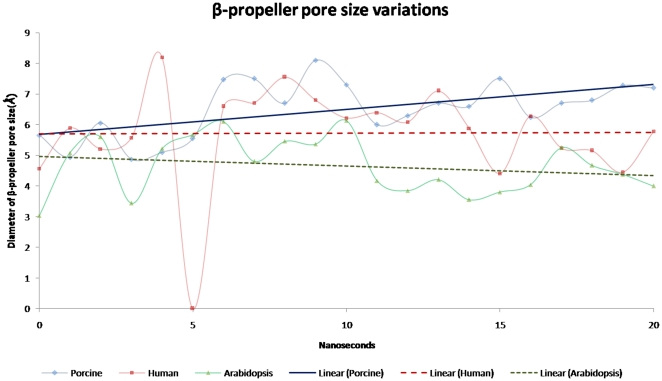
Deviations in β-propeller pore size. During simulation of bound form of porcine, human and *A. thaliana* POP. In porcine diameter of pore was found to be increasing in while human and *A. thaliana* it was found to be decreasing (Figure S7 for replicate runs).

**Figure 7:**
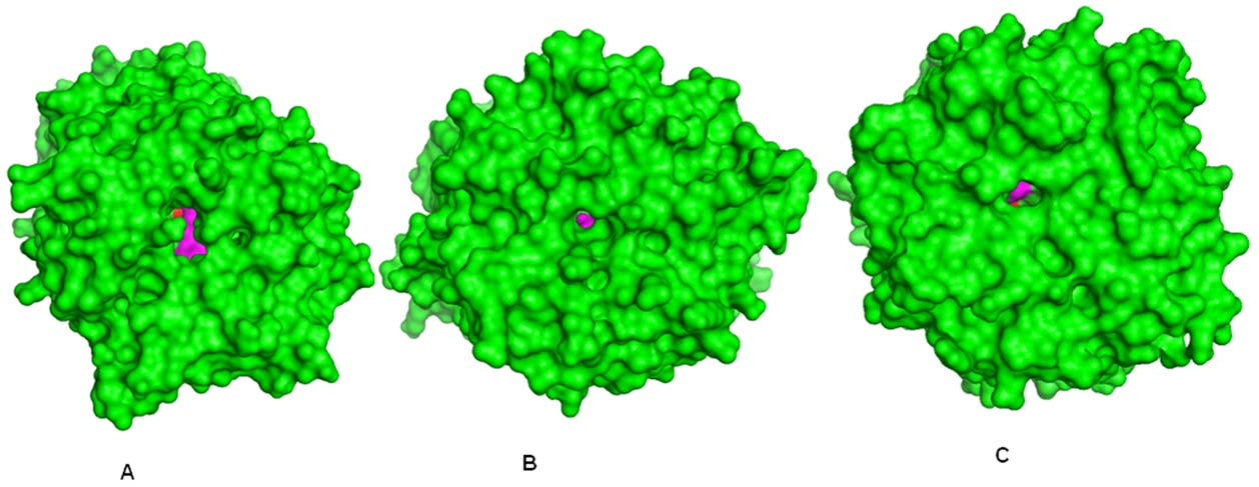
20^th^ ns bound form structure of porcine, human and *A. thaliana* POPs. β-propeller pore size was found to be biggest in porcine, while in human and *A. thaliana* it was small. Drug (ZPR) is shown in magenta color, active site is colored red.

Comparison among three POP species was done with respect to known cavities present in structure to understand the possible passage of substrate entry and product egress. Bound form simulations revealed opening and closing of present cavities of proteins in three POP species. In porcine and human bound form POP during initial 4 ns of simulation, inter-domain cavity (Polgar and coworkers [28]) was present, but after that it was hidden continuously till the end of simulation. Similarly, cavity present in continuation with β-propeller (Tarrago and coworkers [54]) also followed the same trend. On the other hand, in unbound forms, above mentioned cavities were hidden revealing incapability of these cavities in absorbing a substrate and hence its accessibility to active site. Interestingly, we found that β-propeller pore size showed variations in diameter. During simulation, some species-specific striking differences in pore size of β-propeller in three POPs, especially in bound form were noticed. We discovered that in porcine there was continuous increase in pore diameter size from ∼5.5 Å to ∼7.3 Å during simulation while in human (∼4.5 Å to ∼5.0 Å) and *A. thaliana* (∼3.0 Å to 4.2 Å) POP there were not high changes in pore size (Figure 6, Figure 7 and Figure S7). Overall, during simulation β-propeller pore size was increased for porcine, while for human POP it does not show any substantial changes. On the other hand, pore size was decreased continuously for *A. thaliana* POP which shows possibility of some alternate role of β-propeller in plant systems. Pore sizes in unbound forms of POP of human and porcine were smaller in diameter than respective bound forms. Inter-domain distance (Pro34-Thr200) in porcine and human unbound form was increased by 2 Å and 3 Å respectively while *A. thaliana* was also amenable to such changes. This shows domain opening motion while such high differences were not present in bound forms (Table S1). During simulation distance between blade 1 and 7 (Cys78-Glu397) was decreased in both bound/unbound forms of porcine and human suggesting the incapability of movement of blades of propeller (Table S2).

As both domains are found to be connected via series of hydrogen bonds, we checked how hydrogen bonding interactions are changing during simulation including replicate runs. In unbound form, both porcine and *A. thaliana* showed similar trend of loosening of interactions between two domains as number of strong hydrogen bonds were found to be reduced (Table S3) in 20^th^ ns structure, while in human unbound form, more strong bonds were formed which showed its dissimilar trend as compared to porcine and *A. thaliana*. All bound forms were found to be more intact during simulation as shown by increase in number of strong hydrogen bonds during simulation which showed that, in substrate bound condition release of product from opening of two domains is very unlikely. We also noticed changes in strong hydrogen bonds between blade one and seven in the unbound form. Bound form of porcine was more relaxed having less number of strong hydrogen bonds after 20 ns than human and *A. thaliana* POPs.

#### Interplay of lysines present on blade one and seven

**Figure 8:**
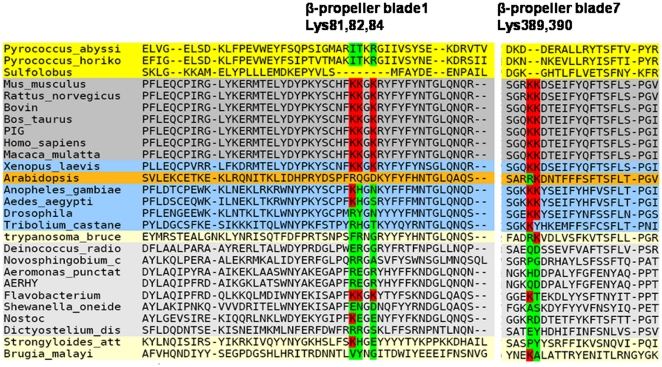
Conservation of lysine residues across lower to higher organisms. Lysines were conserved in all mammalian species and also in amphibian *Xenopus*. Coloring: Archaebacteria (yellow), mammals (dark grey), amphibian (blue), plants (orange), arthropods (blue, below orange), nematodes (light yellow), bacteria (light grey). Presence of lysines are shown using red color, while absence using green color.

**Figure 9:**
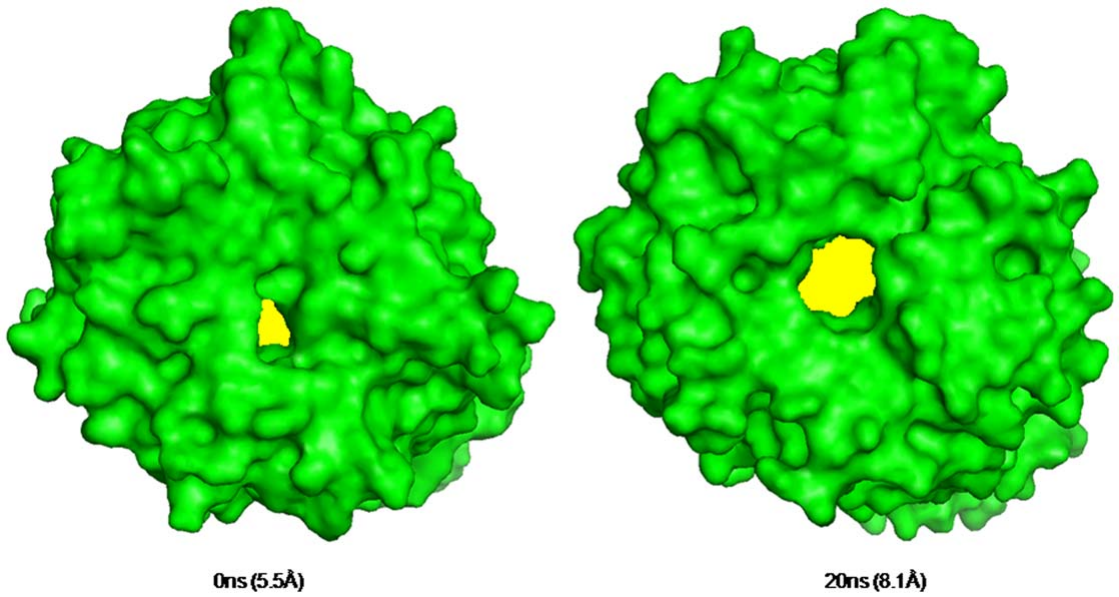
Conformational changes in β-propeller pore size in human POP after *in silico* mutation of lysine residues. Yellow color indicates change in pore size.

During simulation studies of POP we discovered that β-propeller pore was hidden by side chains of lysine residues, three of which are present on blade1 (Lys81, Lys82, Lys84, porcine residue numbering) and two on blade7 (Lys389 and Lys390, porcine residue numbering). Movement of the side chains of these lysines causes fluctuations in β-propeller pore size. Therefore, the conservation of these lysine residues was checked across different species to know if they are present and conserved universally. For this multiple sequence alignment of POPs from different lower to higher organisms (bacteria, archaebacteria, mammals, amphibians, nematodes and plants) was carried out. Interestingly, alignment shows differences in conservation of lysine among different species. From all the species considered for the analysis lysines were totally conserved in mammals and amphibians as shown in Figure 8. In human POP, we observed continuous hindrance in β-propeller pore opening because of side chains of lysines therefore smaller pore size as compared to porcine. This observation leads us to mutate all lysines present on mouth of β-propeller with smaller positively charged histidine. The only intention to mutate lysine with histidine was not to disturb charge distribution on the surface of protein. We found that after mutation pore size was increased to 5.5 Å than wild type (4.5 Å). Further to see dynamics of mutated protein, we subjected the mutant (unbound) to undergo 20 ns simulation like wild type protein. Analysis of trajectory of mutated human POP showed increase in diameter from 5.5 to 8.1 Å (Figure 9). This analysis showed that other species of POP in which lysines are absent and are replaced by smaller amino acids like Ile, Val, Ser etc can have bigger pore size which can lead to differences in substrate entry or product egress. On the other hand if lysines are replaced by longer length amino acids like arginine (e.g. *A. thaliana*) which in turn can contribute in decreasing or completely hiding the β-propeller pore opening.

#### Protein-ligand interactions

Ligand (ZPR)-interacting residues (present within 5 Å from any one of the ligand atoms) were examined, during the course of simulation of the ligand-bound form, to see if changes in interaction with the ligand happen in comparison to the initial structure. Hydrogen bond interactions of ligand with the protein were compared with porcine POP crystal structure. Surprisingly, neither of the species retained the same binding mode as seen in X-ray structure as after 20 ns orientation of the ligand in three POPs was slightly different than the initial structure (Figure S8). Residues present within 5 Å of the binding site can be functionally important as they play an important role in biological activity of the molecule and also take part in biological interactions hence mapping of these interactions was carried out. Hydrogen bonding interactions with Tyr473, Trp595, and Arg641 were observed. Importantly, these interactions between the ligand and interacting residues were not conserved in three POP species (see Table 2 for details). After 20 ns, numbers of hydrogen bonds were found to decrease in porcine and human and we also noticed few new interacting residues coming within 5 Å region of drug. We also noticed differences in interacting hydrophobic residues among the three POPs. Some of the residues, like Ile-591 and Phe-476, retain highly conserved interactions during the 20 ns simulation in both porcine and *A. thaliana* POPs, while only Ile590 was conserved in human POP suggesting that rational mutations of the above residues could be carried out to study the functional importance of these residues.

**Table 2:** Hydrogen bonds at drug binding site (active site) in three POP species.

POP species	Crystal structure/0 ns	Length of H-bond*	After 20 ns	Length of H-bond
**Porcine**				
run1	Tyr-473 [OH]–ZPR-711 [O16]	3.08	Trp-595 [NE1]–ZPR-711 [O2]	2.96
	Tyr-473 [OH]–ZPR-711 [N14]	3.38		
	Trp-595 [NE1]–ZPR-711 [O2]	2.96		
run2	Tyr-643 [OH]–ZPR-711 [O16]	2.70	Arg-643 [NE]–ZPR-711 [O9]	2.55
	Arg-643 [NH1]–ZPR-711 [O9]	2.85	Arg-643 [NH2]–ZPR-711 [O9]	2.65
***A. thaliana***				
run1	**Arg-661 [NH1]–ZPR-732 [O9]**	2.68	Trp-609 [NE1]–ZPR-732 [O9]	3.32
			**Arg-661 [NH1]–ZPR-732 [O9]**	2.84
run2	Trp-609 [NE1]–ZPR-732 [O9]	2.94	**Arg-661 [NH1]–ZPR-732 [O9]**	2.77
	**Arg-661 [NH1]–ZPR-732 [O16]**	2.99		
	Arg-661 [NH1]–ZPR-732 [O9]	2.81		
**Human**				
run1	**Trp-592 [NE1]–ZPR-710 [O16]**	2.16	Trp-592 [NE1]–ZPR-710 [O16]	2.11
	**Arg-640 [NH1]–ZPR-710 [O9]**	3.01		
	Arg-640 [NH1]–ZPR-710 [O2]	3.07		
run2	**Trp-592 [NE1]–ZPR-710 [ O16]**	3.07	Trp-592 [NE1]–ZPR-710 [O16]	3.01
	**Arg-640 [NH1]–ZPR-710 [O9]**	2.93	Arg-640 [NH1]–ZPR-710 [O9]	3.07

Residues in bold indicates same hydrogen bond interactions found during replicate runs.

*H-bonds were defined using a 3.5 Å cutoff distance [H–acceptor] and a 30° cutoff angle [donor–H–acceptor].

#### Simulation from docked poses

**Figure 10:**
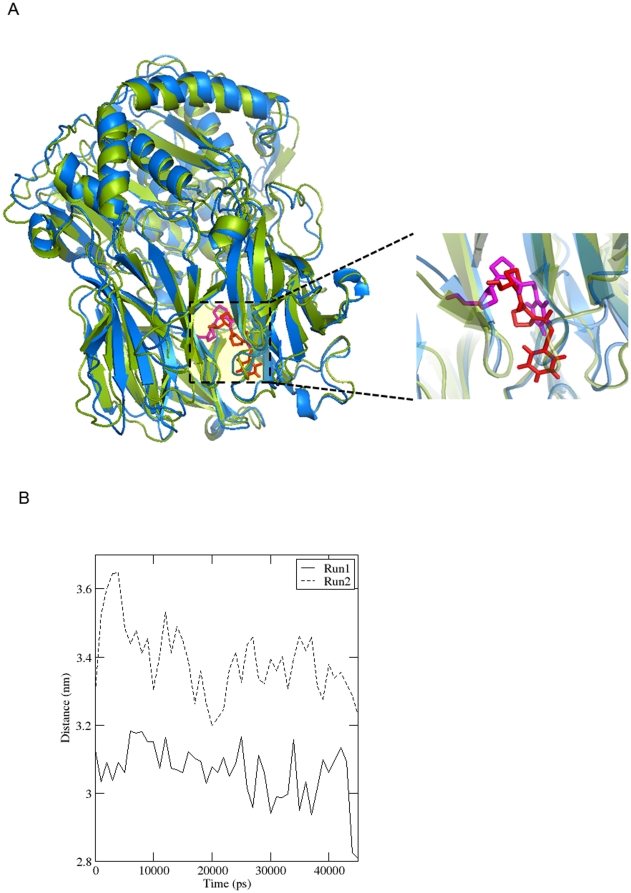
Simulation from blind docking pose where drug was at mouth of β-propeller pore. a) 0^th^ ns structure (protein: green, drug: red) and 20^th^ ns structure (protein: blue, drug: magenta) were superimposed and distance plot of catalytic Ser-554 hydroxyl group and centre of mass of drug was plotted. Distance was found to decrease by more than 2 Å. B) distance plot.

Results of blind docking showed the capability of ligand to bind at multiple sites of POP. So, from blind docking, two docked complexes were selected (firstly, when ligand was at the mouth of β-propeller, secondly, when ligand was at the inter-domain cavity), for molecular dynamics studies to better understand the passage for ligand entry/egress. A 45 ns simulation of porcine POP docked complex was performed, where ligand was at the mouth of β-propeller. Distance plot between centre of mass of drug and nucleophilic Ser554-OH was plotted to see drift towards active site (Figure 10). We observed that distance was reduced to more than 1 Å which depicted potentiality of β-propeller in ingesting a ligand. However, no such distance reduction could be observed when the ligand was in one of the other docked pose (like at inter-domain, recently reported cavity) when 20 ns simulation was carried out. In latter case, distance was decreased in initial few picoseconds only and was constant throughout 20 ns run (data not shown). A distance reduction of >1Å in 45 ns simulations may not be considered significant enough and longer length simulations could be carried out in future to understand the theory of ligand ingestion from β-propeller.

#### Smaller product movement

**Figure 11:**
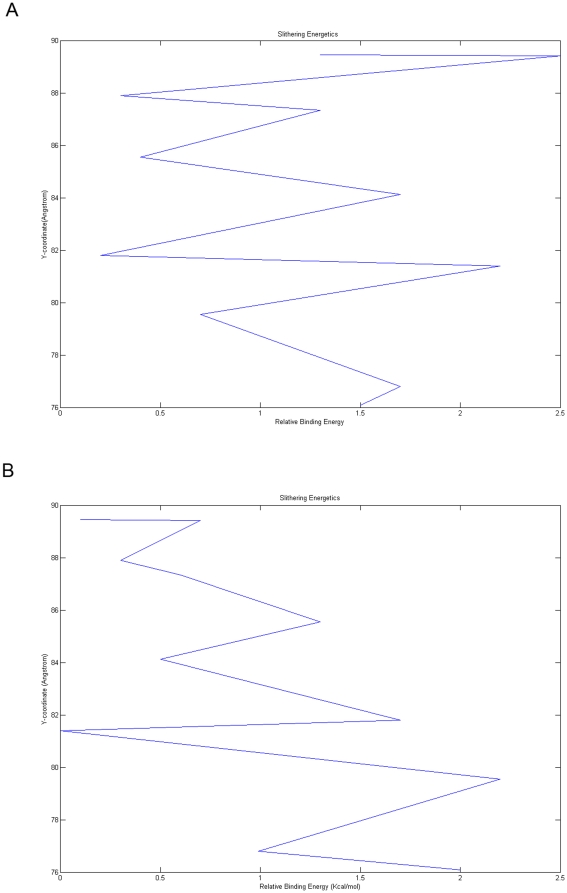
Free energy profile of smaller product movement through β-propeller in porcine POP. a) 0^th^ ns structure which showed huge barrier around β-propeller pore. b) 20^th^ ns structure where free energy barrier was reduced.

Cleavage of any small peptide like angiotensin-IV by POP produces two N- and C-terminal products. SLITHER [76] generates contiguous conformations of ligand across the tunnel. Docking of smaller product in porcine POP (C-terminal) by SLITHER showed that its egress can be through β-propeller pore. Different snapshots generated from molecular dynamics run were used to see movement of C-terminal product from β-propeller pore opening. Further, comparison of free energy profiles was done for different snapshots and it was observed that at 0 ns free energy profile generated showed major barrier at mouth of β-propeller pore while after 20 ns simulated structure having bigger pore size, this barrier was reduced which confirms that smaller product can egress from β-propeller pore (Figure 11). These changes of energetic profiles upon the conformational changes provide an explanation why some conformational changes are beneficial for the entrance or exit of substrate/product.

## Discussion

Differences in structural dynamics among proteins are of key importance which can influence the entire thermodynamics and in turn their biological responses. We have applied molecular docking and dynamics based approaches for exhaustive comparison of prolyl oligopeptidase of three species including porcine, human and *A. thaliana*. POP is an important therapeutic target and is found to be associated with different disorders due to which hundreds of compounds have been tested for POP inhibitory activity. Porcine POP is a well-known model system on which numerous studies have been carried out. Many of POP inhibitors reached preclinical trial stages but till now drugs are not available for public use. This raises the question that to what extent extrapolation of porcine POP to human POP is correct? Plant POPs are poorly understood distant members of same family which diverged a bit earlier than human and porcine POPs. Therefore, this study was carried out to structurally compare porcine, human and plant POPs in detail at two levels: 1) To identify how ligand binding affinities differ among them 2) To compare their structural dynamics which was seen with both bound and unbound forms to envisage the possible path for substrate entry/product egress and to investigate differences among species if any.

Crystal structure and homology model of prolyl oligopeptidase from different species provides an opportunity to examine the differences in structural scenario of three POP species. These POP species were found to have high structural similarities, although plant POPs are evolved before animal POPs and are distant members of same family. Even though sequence and structural similarity among these species are high, their ligand binding affinities are very different. Further, the level of sequence similarities between the three POPs that we chose to study provides a unique opportunity to perform analysis of amino acid changes but in a structural context. At such high sequence identities, the homology models derived are of high quality (Sali and coworkers [77], Figure S10) enough to make gross comparisons of spatially proximate residues and conformational changes as evidenced by molecular dynamics. Moreover, by means of a complete threading of a plant POP on porcine POP template, we are able to compare the sequence-specific changes without dependence on the backbone and overall fold of the protein. We also noticed that some of the ligands were incapable of binding to human POP which indicates high specificity and selectivity of human POP. Moreover, ligand binding was found to be more feasible in human POP. Naturally occurring plant based animal inhibitors like berberine, baicalein were observed to bind well to plant POP which showed their binding capacity but it is yet to characterize whether they are plant POP inhibitors or not, and if yes, how they are localized and regulated in plant system? This is still a fascinating area to be explored in detail.

Based on the detail structural analysis of POPs, we suggest that dynamic conformational changes are associated with the ligand binding in plant and animal POPs. Loosening of interactions between β-propeller and α/β hydrolase domains indicates plants and animal POPs also follow similar trend of domain opening motion for substrate entry as previously observed in bacterial POPs. However, our bound form simulations depicted the intactness of two domains revealing the release of product from some alternate pathway. We investigated that in porcine POP, β-propeller pore size increases in diameter by ∼2 Å indicating smaller product release can be from this route as free energy barrier was reduced. But this increase in β-propeller pore size in bound form was not observed in human and *A. thaliana*. Similarly, analysis of strong hydrogen bond interactions suggests intactness of human POP during both bound and unbound form of simulations. These differences showed difference in free energies for opening motion of two domains in different species. However, we also suspect that these differences in strong hydrogen bonds and β-propeller pore size variation could lead to differences in routes of substrate entry and product release. We found that lysines present on blade one and seven continuously hide the β-propeller pore opening. This effect was more prevalent in human bound form than porcine which leads to broader opening of β-propeller in porcine. It is yet to be figure out that the opening of β-propeller pore is a pH dependent mechanism or not? It is possible that because of the change in pH lysines present on blade one and seven will allow broadening of the annulus of the tunnel and thereby movement of small molecules. In porcine this broad opening of pore acts as a passage for the smaller product movement. But we suspect whether this opening could allow larger product movement? Answering this requires still longer length simulations of both bound and unbound form of proteins. This study also showed that in POPs mode of substrate entry/product egress are via different paths as reported in other POP family members.

Sequence analysis showed that in some species like nematodes, arthropods and even in plants, lysines covering β-propeller pore are absent which would increase diameter of β-propeller pore and eventually can even allow bigger product movement. To our knowledge, this is the first study that reflects role of β-propeller in product movement. As human POP was found to be incapable of binding to few ligands like UAMC and Y-29794, molecular docking with 20^th^ ns structure of human unbound form was re-performed. This showed that after subjecting protein to dynamic state, ligand binding is possible as both the above ligands which showed hindrance in binding were found to bind well due to change in the orientation of the binding as shown in Figure S9 (rigid docking score of UAMC:−5.94 kcal/mol, Y-29794: −4.26 kcal/mol). This depicts that conformational changes are necessary especially at the active site of protein.

This analysis showed differences in conformational dynamics of three POP species with respect to ligand binding and product release. On the whole, the current analysis confirms that the ligand affinities and mode of product egress are different for the plant, porcine and human POPs suggesting that studies with potential drugs have to be carefully interpreted. This work is likely to have implications for the design of inhibitors acting at POP and could contribute to development and maturation of novel leads for neurodegenerative diseases. This analysis will enhance our understanding of evolutionary history of POP protein and will also contribute to understanding of function of the enzyme.

## Supporting Information

Figure S1 **Electrostatic surface potential of β-propeller domain of three species.** a) Porcine b) Human c) *A. thaliana* POPs. The surface between blades 1 and 7 is shown by a black arc for clarity. The blade one of β-propeller is more positively charged in porcine and human as compared to *A. thaliana*. Presence of an extra Arg402 on mouth of β-propeller pore of *A. thaliana* makes it more positive.
(TIF)

Figure S2 **Blind docking results of three POPs using Z-pro prolinal as an inhibitor.** a) porcine b) human c) *A. thaliana* POP. Inhibitor shown in red color was sampled 100 times.
(TIF)

Figure S3 **Blind docking of a dummy molecule.** Blind docking was done to porcine POP to validate whether binding of substrate is random or not. Sucrose was found to have affinity all over the protein while substrate (angiotensin) binding was limited to some expected places. Figures below show comparison of binding poses of sucrose and angiotensin.
(TIF)

Figure S4 **Percentage docked poses of substrate and product.** Docked poses were compared between porcine and human POP a) In porcine POP, out of hundred sampling substrate was not found to bind at inter-domain cavity and also newly reported cavity while product was found to bind all over except inter-domain cavity b) In human POP, substrate has tendency to bind in similar way to porcine POP while product has affinity to bind all over protein.
(TIF)

Figure S5 **Backbone (C^α^) RMSD of two domains.** RMSD of β-propeller (black) and α/β-hydrolase (red) domains separately in porcine POP. RMSD of entire protein (green) is also represented. β-propeller showed higher fluctuations then α/β-hydrolase.
(TIF)

Figure S6 **RMSD of drug present in binding pocket during bound form simulation of porcine and human POP.** a) drug in porcine POP binding pocket b) drug in human POP binding pocket.
(TIF)

Figure S7 **Figure shows β-propeller pore size variations during molecular dynamics replicate runs.** Overall, porcine POP shows increase in β-propeller pore size while human, *A. thaliana* POP these changes were not very high. *A. thaliana* POP shows slight decrease in pore size.
(TIF)

Figure S8 **Superimposed structures of porcine, human and** ***A. thaliana*** **POPs before (0 ns) and after MD (20 ns).** Protein shown in green color is 0 ns structure while in blue color is 20 ns structure. Drug (ZPR) is shown in tint (0 ns) and magenta color (20 ns). Figure shows the difference in orientation of drug after 20 ns in all three species.
(TIF)

Figure S9 **Docking results showing mode of binding of ligands in 0 ns (green) and 20 ns (blue) structures of human POP.** a) UAMC bound to human POP, 0 ns complex is shown green(receptor) and tint (UAMC) color, short contact with Ile-478 is shown in yellow circle b)Y-29794 bound to human POP (b.i) 0 ns structure. (b.ii) 20 ns structure. Residues present in vicinity (4 Å) are shown in yellow color.
(TIF)

Figure S10 **Secondary structure alignment of porcine POP (1E5T) used as a template for building the model structure of** ***A. thaliana*** **.** It clearly shows the agreement of secondary structure elements between two protein sequences. Yellow and pink color indicates beta-strands and alpha helices respectively.
(TIF)

Table S1 **Distance between two domains.** This distance was calculated using Pro34 and Thr200. Distances of replicate runs are shown in brackets.
(DOC)

Table S2 **Distance between blades one and seven during simulation (Cys78 and Glutamine 397).** Distances of replicate runs are shown in brackets.
(DOC)

Table S3 **Residues involved in strong hydrogen bond formation.** a) Number of strong hydrogen bonds during simulations of bound and unbound forms of porcine, human, *A. thaliana* b) Strong inter-domain hydrogen bonds in porcine, human, *A. thaliana* POPs in unbound form. Comparison was done between 0 ns and 20 ns structures c) Strong inter-domain hydrogen bonds in bound form of POP species. Residues in bold indicates same hydrogen bond interactions found during replicate runs.
(DOC)
