# Supplementary figures and images for: Structural Analysis of Prolyl Oligopeptidases Using Molecular Docking and Dynamics: Insights into Conformational Changes and Ligand Binding
Source: PLoS One. 2011 Nov 23;6(11):e26251. doi: 10.1371/journal.pone.0026251 (PMC3223163; doi:10.1371/journal.pone.0026251)

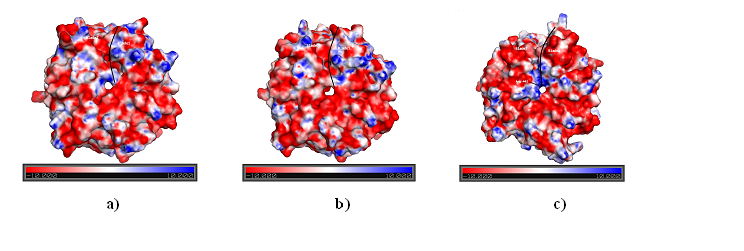

Supplement: Figure S1 — Electrostatic surface potential of β-propeller domain of three species. a) Porcine b) Human c) A. thaliana POPs. The surface between blades 1 and 7 is shown by a black arc for clarity. The blade one of β-propeller is more positively charged in porcine and human as compared to A. thaliana. Presence of an extra Arg402 on mouth of β-propeller pore of A. thaliana makes it more positive. (TIF) [file pone.0026251.s001.tif]

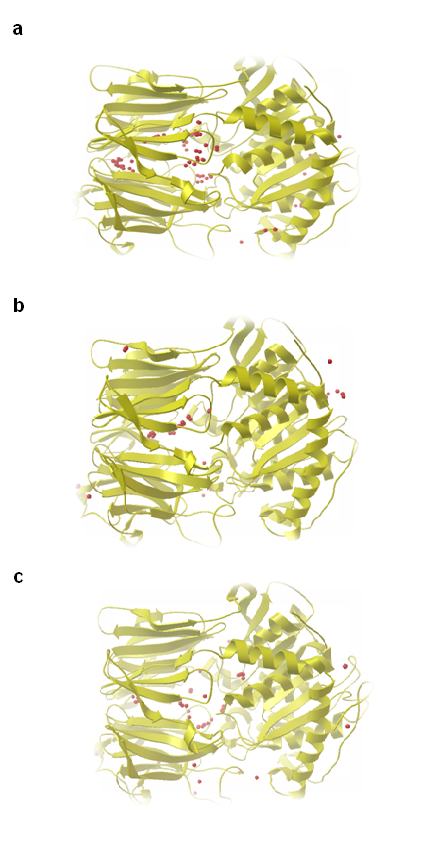

Supplement: Figure S2 — Blind docking results of three POPs using Z-pro prolinal as an inhibitor. a) porcine b) human c) A. thaliana POP. Inhibitor shown in red color was sampled 100 times. (TIF) [file pone.0026251.s002.tif]

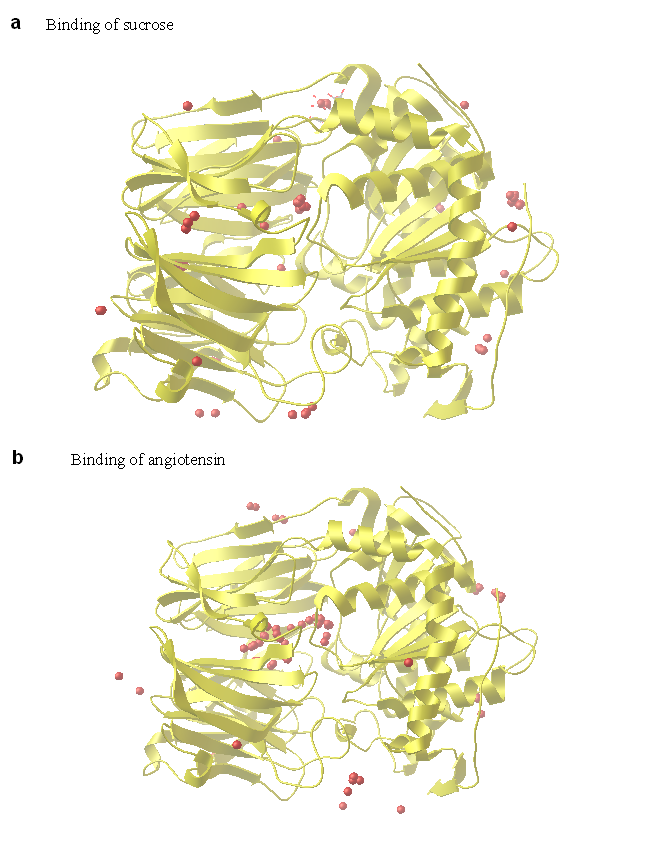

Supplement: Figure S3 — Blind docking of a dummy molecule. Blind docking was done to porcine POP to validate whether binding of substrate is random or not. Sucrose was found to have affinity all over the protein while substrate (angiotensin) binding was limited to some expected places. Figures below show comparison of binding poses of sucrose and angiotensin. (TIF) [file pone.0026251.s003.tif]

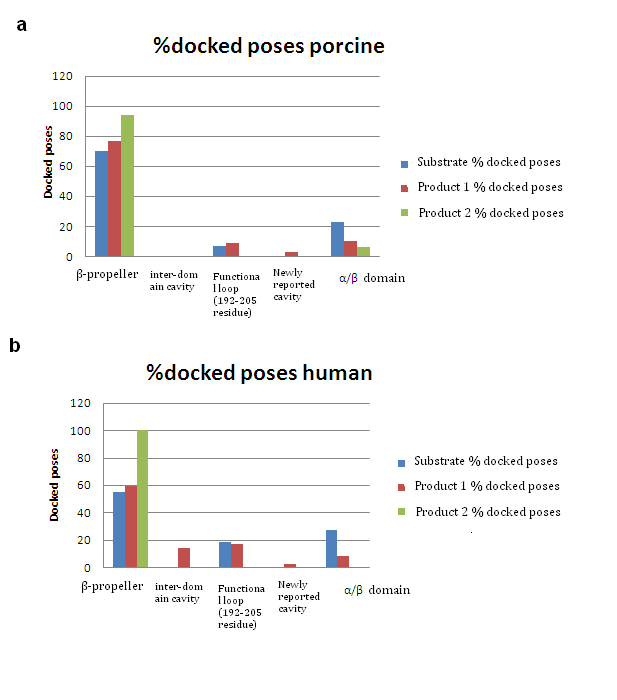

Supplement: Figure S4 — Percentage docked poses of substrate and product. Docked poses were compared between porcine and human POP a) In porcine POP, out of hundred sampling substrate was not found to bind at inter-domain cavity and also newly reported cavity while product was found to bind all over except inter-domain cavity b) In human POP, substrate has tendency to bind in similar way to porcine POP while product has affinity to bind all over protein. (TIF) [file pone.0026251.s004.tif]

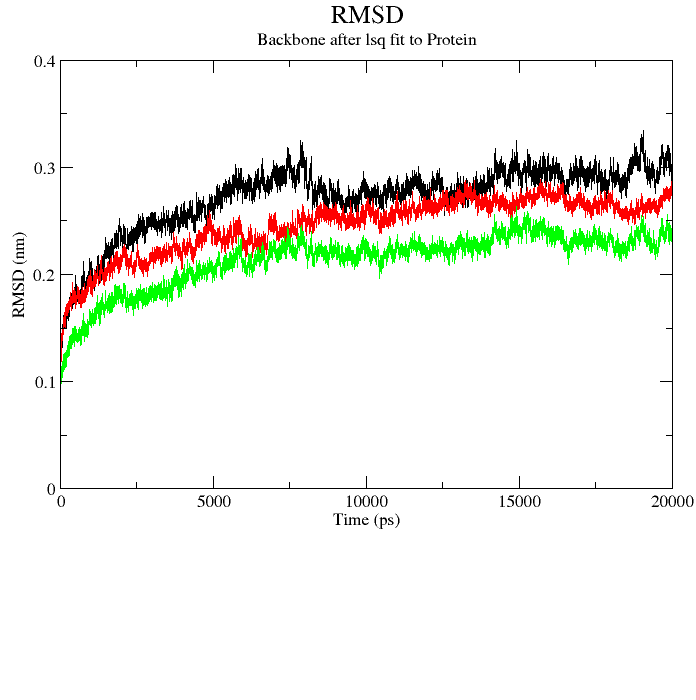

Supplement: Figure S5 — Backbone (Cα) RMSD of two domains. RMSD of β-propeller (black) and α/β-hydrolase (red) domains separately in porcine POP. RMSD of entire protein (green) is also represented. β-propeller showed higher fluctuations then α/β-hydrolase. (TIF) [file pone.0026251.s005.tif]

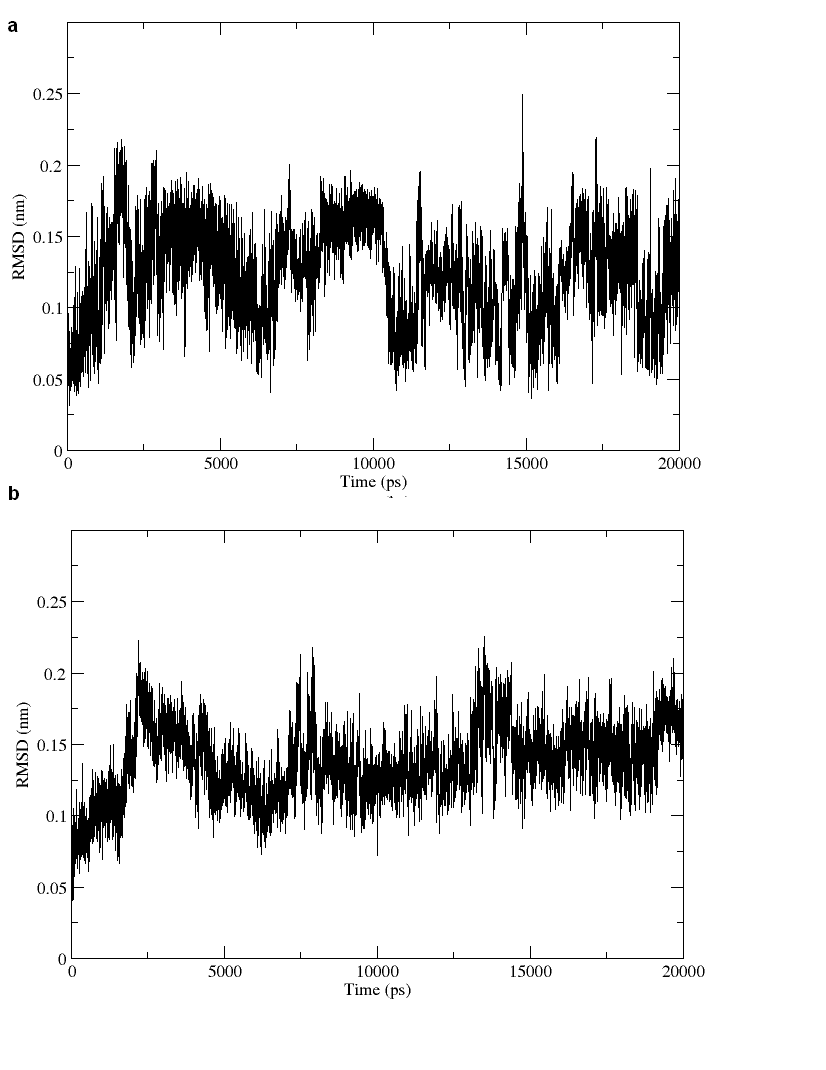

Supplement: Figure S6 — RMSD of drug present in binding pocket during bound form simulation of porcine and human POP. a) drug in porcine POP binding pocket b) drug in human POP binding pocket. (TIF) [file pone.0026251.s006.tif]

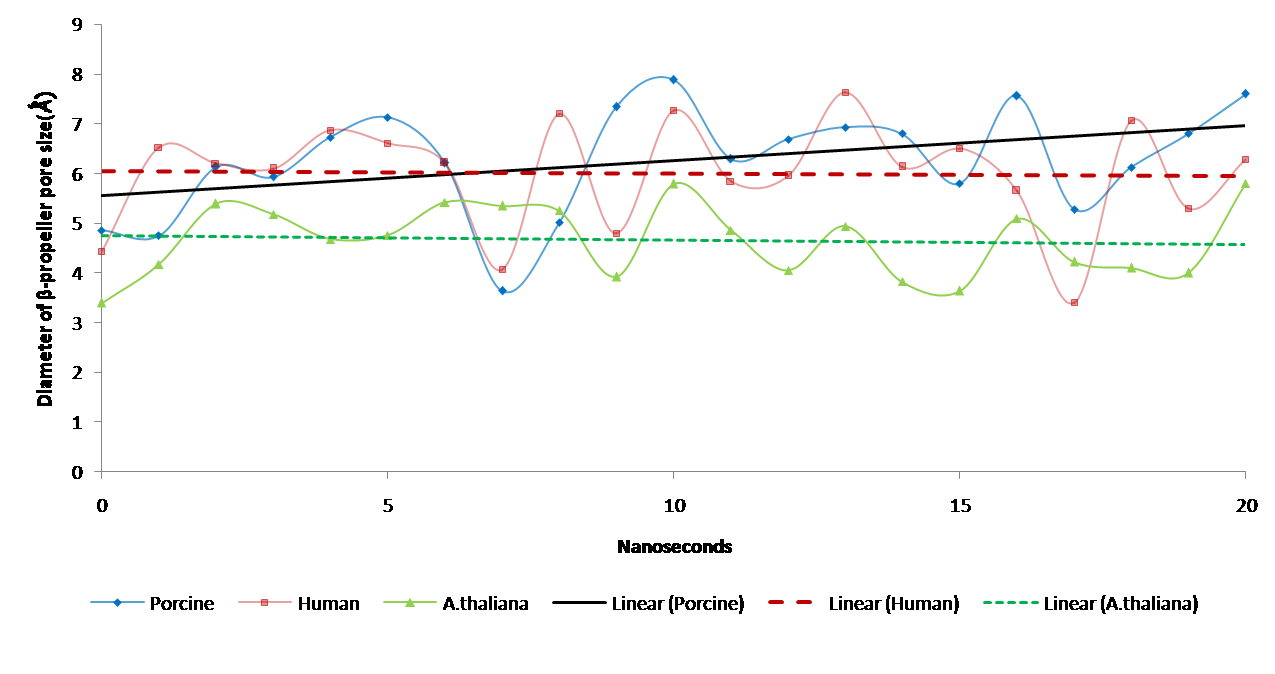

Supplement: Figure S7 — Figure shows β-propeller pore size variations during molecular dynamics replicate runs. Overall, porcine POP shows increase in β-propeller pore size while human, A. thaliana POP these changes were not very high. A. thaliana POP shows slight decrease in pore size. (TIF) [file pone.0026251.s007.tif]

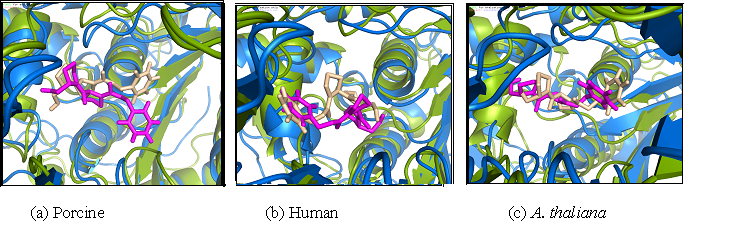

Supplement: Figure S8 — Superimposed structures of porcine, human and A. thaliana POPs before (0 ns) and after MD (20 ns). Protein shown in green color is 0 ns structure while in blue color is 20 ns structure. Drug (ZPR) is shown in tint (0 ns) and magenta color (20 ns). Figure shows the difference in orientation of drug after 20 ns in all three species. (TIF) [file pone.0026251.s008.tif]

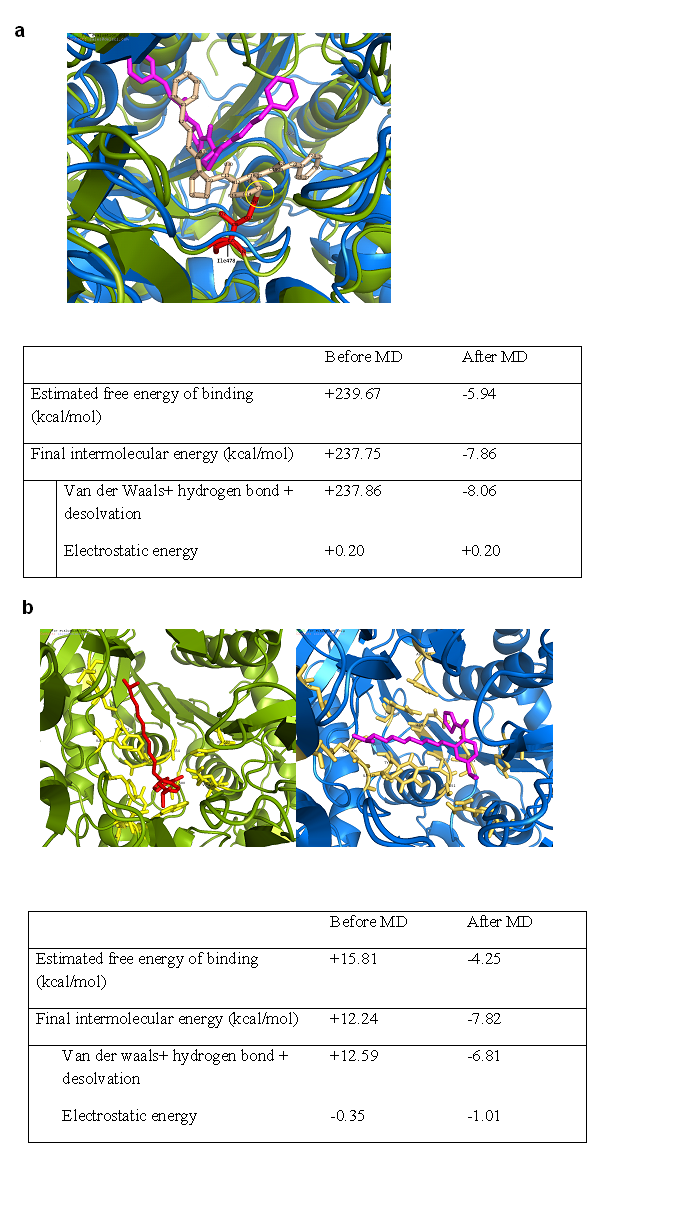

Supplement: Figure S9 — Docking results showing mode of binding of ligands in 0 ns (green) and 20 ns (blue) structures of human POP. a) UAMC bound to human POP, 0 ns complex is shown green(receptor) and tint (UAMC) color, short contact with Ile-478 is shown in yellow circle b)Y-29794 bound to human POP (b.i) 0 ns structure. (b.ii) 20 ns structure. Residues present in vicinity (4 Å) are shown in yellow color. (TIF) [file pone.0026251.s009.tif]

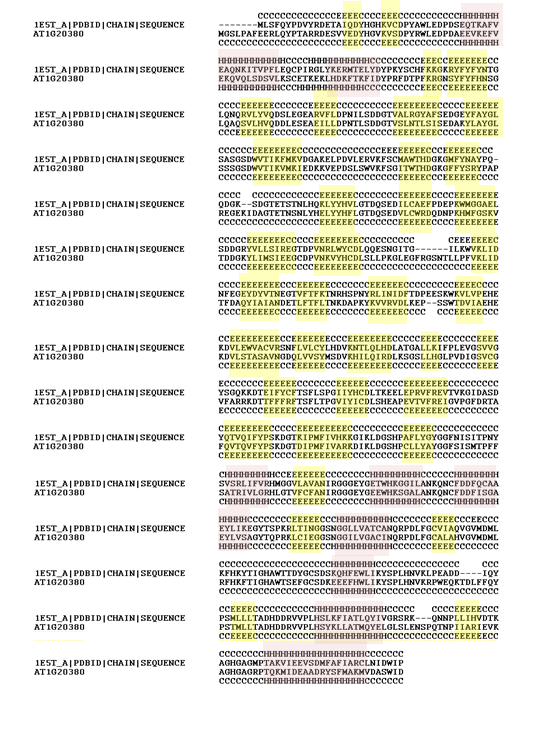

Supplement: Figure S10 — Secondary structure alignment of porcine POP (1E5T) used as a template for building the model structure of A. thaliana . It clearly shows the agreement of secondary structure elements between two protein sequences. Yellow and pink color indicates beta-strands and alpha helices respectively. (TIF) [file pone.0026251.s010.tif]
