# Supplementary material for: Structural Analysis of Prolyl Oligopeptidases Using Molecular Docking and Dynamics: Insights into Conformational Changes and Ligand Binding
Source: PLoS One. 2011 Nov 23;6(11):e26251. doi: 10.1371/journal.pone.0026251 (PMC3223163; doi:10.1371/journal.pone.0026251)
Supplement: Table S1 — Distance between two domains. This distance was calculated using Pro34 and Thr200. Distances of replicate runs are shown in brackets. (DOC) [file pone.0026251.s011.doc]

**Table S1: Distance between two domains. This distance was calculated using Pro34 and Thr200.** Distances of replicate runs are shown in brackets.

| **POPs** | **Bound** | | **Unbound** | |
| --- | --- | --- | --- | --- |
|  | 0ns | 20ns | 0ns | 20ns |
| **Human** | 7 (7.5) | 8.5 (9.8) | 6.9 (6.9) | 10.4 (10.1) |
| **Porcine** | 7 (6.8) | 7.9 (8.1) | 6.9 (7.2) | 8.9 (10.9) |
| ***A. thaliana*** | 6.7 (6.8) | 7.6 (5.5) | 7.0 (7.0) | 7.0 (15.0) |
