# Supplementary material for: Structural Analysis of Prolyl Oligopeptidases Using Molecular Docking and Dynamics: Insights into Conformational Changes and Ligand Binding
Source: PLoS One. 2011 Nov 23;6(11):e26251. doi: 10.1371/journal.pone.0026251 (PMC3223163; doi:10.1371/journal.pone.0026251)
Supplement: Table S2 — Distance between blades one and seven during simulation (Cys78 and Glutamine 397). Distances of replicate runs are shown in brackets. (DOC) [file pone.0026251.s012.doc]

**Table S2: Distance between blades one and seven during simulation (Cys78 and Glutamine 397).** Distances of replicate runs are shown in brackets.

| **POPs** | **Bound** | | **Unbound** | |
| --- | --- | --- | --- | --- |
|  | 0ns | 20ns | 0ns | 20ns |
| **Human** | 6.8 (6.4) | 6.3 (7.8) | 6.6 (6.6) | 6.3 (5.7) |
| **Porcine** | 6.5 (6.4) | 6.5 (6.8) | 6.6 (6.3) | 5.7 (6.0) |
| ***A. thaliana*** | 5.8 (5.7) | 6.2 (5.7) | 5.8 (5.8) | 6.2 (6.3) |
