# Supplementary material for: Structural Analysis of Prolyl Oligopeptidases Using Molecular Docking and Dynamics: Insights into Conformational Changes and Ligand Binding
Source: PLoS One. 2011 Nov 23;6(11):e26251. doi: 10.1371/journal.pone.0026251 (PMC3223163; doi:10.1371/journal.pone.0026251)
Supplement: Table S3 — Residues involved in strong hydrogen bond formation. a) Number of strong hydrogen bonds during simulations of bound and unbound forms of porcine, human, A. thaliana b) Strong inter-domain hydrogen bonds in porcine, human, A. thaliana POPs in unbound form. Comparison was done between 0 ns and 20 ns structures c) Strong inter-domain hydrogen bonds in bound form of POP species. Residues in bold indicates same hydrogen bond interactions found during replicate runs. (DOC) [file pone.0026251.s013.doc]

**Table S3: Residues involved in strong hydrogen bond formation** a) Number of strong hydrogen bonds during simulations of bound and unbound forms of porcine, human, *A. thaliana* b) Strong inter-domain hydrogen bonds in porcine, human, *A. thaliana* POPs in unbound form. Comparison was done between 0ns and 20ns structures c) Strong inter-domain hydrogen bonds in bound form of POP species. Residues in bold indicates same hydrogen bond interactions found during replicate runs.

a)

| **Species** | **Bound** | | | | **Unbound** | | | |
| --- | --- | --- | --- | --- | --- | --- | --- | --- |
|  | Run1 | | Run2 | | Run1 | | Run2 | |
|  | 0ns | 20ns | 0ns | 20ns | 0ns | 20ns | 0ns | 20ns |
| **Porcine** | 11 | 15 | 13 | 17 | 16 | 12 | 16 | 15 |
| **Human** | 10 | 17 | 13 | 25 | 10 | 15 | 10 | 10 |
| ***A. thaliana*** | 11 | 16 | 16 | 17 | 17 | 13 | 19 | 13 |

b)

|  | **0ns** | | | **20ns** | | |
| --- | --- | --- | --- | --- | --- | --- |
|  | β-propeller | α/β-hydrolase | Distance (Ǻ) | β-propeller | α/β-hydrolase | Distance (Ǻ) |
| **Porcine** | **Gln-95 (O)** | **Thr-686 (OG1)** | 2.54 | **Leu-94 (N)** | **Asp-72 (OD1)** | 2.15 |
| **run1** | **Asn-96 (OD1)** | **Ala-687 (N)** | 1.98 | Asp-122 (O) | Thr-686 (N) | 1.83 |
|  | Gln-97 (NE2) | Gly-683 (O) | 2.79 | Ala-145 (O) | Gly-683 (N) | 2.13 |
|  | **Ser-148 (OG)** | **Asp-642 (OD1)** | 2.66 | **Asp-149 (N)** | **Asp-642 (OD2)** | 2.03 |
|  | Ser-148 (OG) | Asp-642 (O) | 2.67 | Glu-201 (O) | Thr-590 (N) | 1.91 |
|  | **Asp-149 (OD2)** | **Arg-643 (NE)** | 2.01 | **Asn-205 (N)** | **Thr-590 (O)** | 1.81 |
|  | **Thr-202 (O)** | **Thr-590 (N)** | 2.11 | Cys-255 (N) | His-593 (O) | 1.99 |
|  | Thr-204 (OG1) | His-593 (ND1) | 2.17 | Asp-256 (OD1) | Thr-597 (OG1) | 2.71 |
|  | **Asn-205 (N)** | **Thr-590 (O)** | 1.95 | **Pro-309 (O)** | **Glu-512 (N)** | 2.16 |
|  | Cys-255 (N) | Thr-597 (OG1) | 2.03 | Asp-356 (OD1) | Tyr-510 (OH) | 2.67 |
|  | Asp-256 (N) | Thr-597 (OG1) | 2.29 | Asp-356 (N) | Tyr-510 (OH) | 2.66 |
|  | **Pro-309 (O)** | **Glu-512 (N)** | 2.16 | Ser-381 (OG) | His-593 (O) | 2.73 |
|  | **Asp-356 (OD1)** | **Tyr-510 (OH)** | 2.63 |  |  |  |
|  | Asp-356 (N) | Tyr-510 (OH) | 2.7 |  |  |  |
|  | **Val-427 (O)** | **Ile-430 (N)** | 2.36 |  |  |  |
|  | Thr-124 (OG1) | Lys-677 (NZ) | 2.79 |  |  |  |
| **run2** | **Asn-205 (N)** | **Thr-590 (O)** | 1.85 | **Asn-205(N)** | **Thr-590 (O)** | 1.68 |
|  | Asp-149 (N) | Asp-642 (OD2) | 1.88 | **Pro-309 (O)** | **Glu-512 (N)** | 2.01 |
|  | **Asp-149 (OD2)** | **Arg-643 (NE)** | 2.04 | Glu-201 (N) | Asp-641 (O) | 2.02 |
|  | **Asn-96 (OD1)** | **Ala-687 (N)** | 2.06 | Tyr-190 (OH) | His-593 (NE2) | 2.1 |
|  | Asn-96 (O) | Thr-686 (N) | 2.16 | Ser-148 (N) | Ala-682 (O) | 2.13 |
|  | **Pro-309 (O)** | **Glu-512 (N)** | 2.23 | Gly-147 (O) | Gly-679 (N) | 2.14 |
|  | **Val-427 (O)** | **Ile-430 (N)** | 2.36 | **Leu-94 (N)** | **Asp-72 (OD2)** | 2.24 |
|  | **Thr-202 (O)** | **Thr-590 (N)** | 2.42 | Ser-381 (N) | Pro-482 (O) | 2.26 |
|  | Trp-150 (N) | Asp-642 (OD1) | 2.4 | **Asp-149 (N)** | **Asp-642 (OD2)** | 2.29 |
|  | His-593 (ND1) | Thr-204 (OG1) | 2.44 | Thr-202 (O) | Thr-590 (N) | 2.3 |
|  | **Gln-95 (O)** | **Thr-686 (OG1)** | 2.57 | Thr-399 (OG1) | Tyr-484 (N) | 2.49 |
|  | **Asp-356 (OD1)** | **Tyr-510 (OH)** | 2.77 | Thr-200 (OG1) | Asp-639 (O) | 2.5 |
|  | Tyr-510 (OH) | Asp-356 (OD1) | 2.77 | Glu-339 (OE1) | Ser-479 (OG) | 2.65 |
|  | **Ser-148 (OG)** | **Asp-642 (OD1)** | 2.79 | Asn-96 (ND2) | Lys-684 (O) | 2.72 |
|  | **Ser-148 (OG)** | **Asp-642 (OD1)** | 2.79 | Leu-94 (O) | Thr-686 (OG1) | 2.73 |
|  | Asp-356 (O) | Gln-439 (NE2) | 2.8 |  |  |  |
|  |  |  |  |  |  |  |
| **Human** | Leu-93 (N) | Asp-71 (OD1) | 2.31 | Asp-71 (OD2) | Leu-93 (N) | 1.99 |
| **run1** | Gln-94 (O) | Thr-683 (OG1) | 2.58 | Ser-147 (N) | Asp-638 (OD1) | 2.2 |
|  | **Asn-95 (OD1)** | **Ala-684 (N)** | 1.96 | Ser-147 (OG) | Asp-639 (OD2) | 2.72 |
|  | Asn-95 (O) | Thr-683 (N) | 1.99 | Ser-147 (OG) | Asp-639 (OD2) | 2.72 |
|  | **Asn-204 (N)** | **Thr-587 (O)** | 1.98 | Glu-200 (O) | Thr-587 (OG1) | 2.69 |
|  | **Cys-254 (N)** | **Ala-591 (O)** | 1.87 | **Asn-204 (N)** | **Thr-587 (O)** | 1.93 |
|  | **Pro-308 (O)** | **Glu-509 (N)** | 1.88 | Cys-254 (N) | Thr-594 (OG1) | 2.47 |
|  | Ser-380 (OG) | Pro-479 (O) | 2.67 | Cys-254 (O) | Asn-474 (ND2) | 2.65 |
|  | **Thr-398 (OG1)** | **Tyr-481 (N)** | 2.41 | **Pro-308 (O)** | **Glu-509 (N)** | 1.98 |
|  | Val-425 (O) | Ile-427 (N) | 2.46 | Glu-338 (OE2) | Ser-476 (OG) | 2.74 |
|  |  |  |  | Glu-338 (OE2) | Ser-476 (OG) | 2.74 |
|  |  |  |  | Ser-380 (OG) | Asn-480 (ND2) | 2.74 |
|  |  |  |  | Ser-380 (OG) | Thr-478 (OG1) | 2.77 |
|  |  |  |  | Ser-380 (OG) | Thr-478 (O) | 2.34 |
|  |  |  |  | Thr-398 (OG1) | Tyr-481 (N) | 2.39 |
| **run2** | **Cys-254(N)** | **Ala-591(O)** | 1.81 | **Asn-204 (N)** | **Thr-587 (O)** | 1.73 |
|  | Asn-04(O) | His-590 (NE2) | 1.93 | Thr-201(O) | Thr-587 (N) | 2.06 |
|  | **Asn-95 (OD1)** | **Ala-684 (N)** | 1.94 | Ser-399 (O) | Tyr-481(N) | 2.07 |
|  | **Asn-204 (N)** | **Thr-587 (O)** | 1.98 | Leu-93 (N) | Asp-71(OD2) | 2.09 |
|  | Asp-148 (N) | Asp-639 (OD1) | 2.01 | Asp-148 (N) | Asp-639 (OD1) | 2.1 |
|  | **Pro-308 (O)** | **Glu-509 (N)** | 2.09 | Asn-204 (O) | His-590 (NE2) | 2.12 |
|  | Asn-95 (O) | Thr-683 (N) | 2.1 | **Pro-308 (O)** | **Glu-509 (N)** | 2.14 |
|  | Val-425 (O) | Ile-427 (N) | 2.39 | Asp-355 (OD1) | Tyr-507 (OH) | 2.76 |
|  | **Thr-398 (OG1)** | **Tyr-481 (N)** | 2.41 | Asp-355(OD1) | Tyr-507 (OH) | 2.76 |
|  | Ser-380 (OG) | Thr-478 (OG1) | 2.56 | Asn-304 (O) | Glu-509 (OE2) | 2.8 |
|  |  |  |  |  |  |  |
| ***A. thaliana*** | **Leu-101 (N)** | **Asp-79 (OD1)** | 2.38 | Gln-102 (O) | Thr-707 (OG1) | 2.66 |
| **run1** | **Gln-102 (O)** | **Thr-707 (OG1)** | 2.6 | Ser-153 (OG) | Gly-700 (N) | 2.44 |
|  | Ala-102 (O) | Thr-707 (N) | 2.15 | Gly-209 (O) | Asp-659 (N) | 2.12 |
|  | Asp-129 (OD2) | Lys-698 (NZ) | 2.97 | **Glu-211 (O)** | **Thr-604 (N)** | 2.26 |
|  | **Ser-133 (OG)** | **Gly-704 (O)** | 2.59 | **Asn-215 (N)** | **Thr-604 (O)** | 2.08 |
|  | Asp-156 (OD2) | Arg-661 (NE) | 1.95 | Asn-215 (O) | His-607 (NE2) | 2.35 |
|  | **Trp-157 (N)** | **Asp-660 (OD2)** | 2.25 | Cys-265 (N) | Ala-608 (O) | 1.76 |
|  | **Thr-212 (O)** | **Thr-604 (N)** | 1.84 | Pro-325 (O) | Glu-527 (N) | 2.2 |
|  | **Asn-215 (N)** | **Thr-604 (O)** | 1.8 | Pro-325 (O) | Glu-526 (N) | 2.24 |
|  | Asn-215 (O) | His-607 (NE2) | 2.18 | Asp-370 (OD1) | Tyr-524 (OH) | 2.75 |
|  | Cys-265 (N) | Ala-608 (O) | 2.41 | Asp-370 (OD2) | Tyr-524 (OH) | 2.35 |
|  | Cys-265 (N) | Ser-611 (OG) | 2.02 | Ser-395 (N) | Pro-496 (O) | 2.29 |
|  | **Asp-266 (OD1)** | **Ser-611 (OG)** | 2.72 | Ser-414 (O) | Phe-498 (N) | 2.07 |
|  | Asp-266 (OD2) | Ser-611 (OG) | 2.77 |  |  |  |
|  | **Pro-325 (O)** | **Glu-526 (N)** | 1.97 |  |  |  |
|  | Lys-372 (NZ) | Gln-453 (OE1) | 2.72 |  |  |  |
|  | **Ser-395 (OG)** | **Thr-495 (OG1)** | 2.69 |  |  |  |
| **run2** | **Asn-215(N)** | **Thr-604(O)** | 1.67 | Asp-156 (N) | Asp-660 (OD2) | 1.81 |
|  | Cys-65(N) | Ser-611(OG) | 1.84 | Lys-326 (N) | Glu-526 (OE1) | 2.01 |
|  | Asp-156(OD2) | Arg-661(NE) | 1.89 | Ser-214 (N) | Lys-602 (O) | 2.02 |
|  | **Thr-212(O)** | **Thr-604(N)** | 2 | Val-371 (N) | Tys-524 (OH) | 2.08 |
|  | Asp-56(N) | Asp-660(OD1) | 2.05 | **Asn-215 (N)** | **Thr-604 (O)** | 2.11 |
|  | **Pro-325(O)** | **Glu-526(N)** | 2.1 | **Glu-211 (O)** | **Thr-604 (N)** | 2.26 |
|  | **Leu-101(N)** | **Asp-79(OD1)** | 2.23 | Cys-265 (N) | His-607 (O) | 2.3 |
|  | Ala-103(O) | Thr-707(N) | 2.23 | Ala-103 (O) | Thr-707 (N) | 2.34 |
|  | Ser-414(O) | Phe-498(N) | 2.31 | Cys-265 (N) | Thr-10 (OG1) | 2.49 |
|  | Thr-413(OG1) | Phe-498(N) | 2.37 | Ser-395 (OG) | Thr-495 (OG1) | 2.55 |
|  | **Trp-157(N)** | **Asp-660(OD2)** | 2.46 | Ser-155 (OG) | Asp-660 (OD2) | 2.66 |
|  | Ser-395(OG) | Pro-496(O) | 2.57 | Ser-155 (OG) | Asp-660 (OD2) | 2.66 |
|  | Asp-370(OD1) | Tyr-524(OH) | 2.58 | Thr-210 (OG1) | Asp-659(O) | 2.71 |
|  | Asp-370(OD1) | Tyr-524(OH) | 2.58 |  |  |  |
|  | **Ser-395(OG)** | **Thr-495(OG1)** | 2.58 |  |  |  |
|  | **Asp-266(OD1)** | **Ser-611(OG)** | 2.59 |  |  |  |
|  | **Gln-102(O)** | **Thr-707(OG1)** | 2.72 |  |  |  |
|  | Tyr-80(N) | Ile-78(O) | 2.76 |  |  |  |
|  | **Ser-133(OG)** | **Gly-704(O)** | 2.77 |  |  |  |

c)

|  | **0ns** | | | **20ns** | | |
| --- | --- | --- | --- | --- | --- | --- |
|  | β-propeller | α/β-hydrolase | Distance (Ǻ) | β-propeller | α/β-hydrolase | Distance (Ǻ) |
| **Porcine** | **Gln-95 (O)** | **Thr-686 (OG1)** | 2.7 | Leu-94 (N) | Asp-72 (OD2) | 1.96 |
| **run1** | Asn-96 (OD1) | Ala-687 (N) | 1.79 | Leu-94 (O) | Thr-686 (OG1) | 2.76 |
|  | Gln-97 (NE2) | Gly-683 (O) | 2.75 | Thr-124 (O) | Gly-683 (N) | 2.13 |
|  | **Asp-149 (N)** | **Asp-642 (OD2)** | 2.01 | Ser-144 (OG) | Ala-682 (N) | 2.49 |
|  | **Asp-149 (OD2)** | **Arg-643 (NE)** | 2.13 | Gly-147 (O) | Gly-679 (N) | 1.88 |
|  | **Thr-202 (O)** | **Thr-590 (N)** | 2.06 | Asp-149 (N) | Asp-642 (OD1) | 2.16 |
|  | Asn-205 (N) | Thr-590 (O) | 1.68 | Gly-199 (O) | Asp-641 (N) | 1.97 |
|  | **Cys-255 (N)** | **Thr-597 (OG1)** | 2.12 | Thr-200 (N) | Asp-33 (OD1) | 1.82 |
|  | **Pro-309 (O)** | **Glu-512 (N)** | 2.12 | Glu-201 (O) | Thr-590 (N) | 2.11 |
|  | Asp-356 (OD1) | Tyr-510 (OH) | 2.69 | Thr-204 (N) | Lys-588 (O) | 2.31 |
|  | Ser-381 (OG) | Pro-482 (O) | 2. 69 | Cys-255 (N) | Thr-597 (OG1) | 1.98 |
|  |  |  |  | **Ser-308 (N)** | **Glu-512 (OE2)** | 2.02 |
|  |  |  |  | **Asp-356 (OD2)** | **Tyr-510 (OH)** | 2.68 |
|  |  |  |  | Asp-356 (OD2) | Tyr-510 (OH) | 2.68 |
|  |  |  |  | Thr-399 (OG1) | Tyr-484 (N) | 2.09 |
| **run2** | Asn-205(N) | Thr-590(O) | 1.63 | Asn-205(N) | Thr-590(O) | 1.78 |
|  | **Asp-149(N)** | **Asp-642(OD2)** | 1.9 | **Ser-308(N)** | **Glu-512(OE1)** | 1.84 |
|  | **Asp-149(OD2)** | **Arg-643(NE)** | 2.05 | Pro-309(O) | Glu-512(N) | 1.9 |
|  | **Pro-309(O)** | **Glu-512(N)** | 2.09 | Glu-201(N) | Asp-642(OD1) | 2.02 |
|  | **Cys-255(N)** | **Thr-597(OG1)** | 2.2 | Asn-96(OD1) | Thr-686(N) | 2.05 |
|  | Val-427(O) | Ile-430(N) | 2.32 | Thr-399(OG1) | Tyr-484(N) | 2.32 |
|  | Asn-96(O) | Thr-686(N) | 2.39 | Asp-256(N) | Thr-597(OG1) | 2.47 |
|  | **Gln-95(O)** | **Thr-686(OG1)** | 2.4 | Asn-96(OD1) | Thr-686(OG1) | 2.66 |
|  | **Thr-202(O)** | **Thr-590(N)** | 2.4 | Glu-339(OE2) | Ser-479(OG) | 2.67 |
|  | Asp-56(OD1) | Tyr-510(OH) | 2.67 | Leu-94(O) | Thr-686(OG1) | 2.7 |
|  | Asp-56(OD1) | Tyr-510(OH) | 2.67 | **Asp-356(OD1)** | **Tyr-510(OH)** | 2.74 |
|  | Ser-148(OG) | Asp-642(OD1) | 2.74 | Ser-148(OG) | Asp-642(OD2) | 2.78 |
|  | Ser-148(OG) | Asp-642(OD1) | 2.74 | Cys-255(O) | Asn-477(ND2) | 3.18 |
|  |  |  |  | Asn-305(O) | Glu-512(OE1) | 3.33 |
|  |  |  |  | Glu-253(OE2) | His-593(O) | 3.4 |
|  |  |  |  | Ser-381(OG) | Thr-481(OG1) | 3.43 |
|  |  |  |  | Glu-339(OE1) | Ser-479(OG) | 3.46 |
|  |  |  |  |  |  |  |
| **Human** | Leu-93 (N) | Asp-71 (OD1) | 2.2 | Tyr-72 (N) | Tyr-70 (O) | 2.75 |
| **run1** | **Gln-94 (O)** | **Thr-683 (OG1)** | 2.68 | Asn-95 (OD1) | Ala-684 (N) | 1.89 |
|  | **Asn-95 (OD1)** | **Ala-684 (N)** | 1.86 | Gly-198 (N) | His-637 (ND1) | 2.42 |
|  | **Asn-95 (O)** | **Thr-683 (N)** | 1.92 | Asn-204 (N) | Thr-587 (O) | 2.17 |
|  | **Asn-204 (N)** | **Thr-587 (O)** | 1.98 | **Asn-204 (O)** | **His-590 (NE2)** | 2.33 |
|  | Asn-204 (O) | His-590 (NE2) | 2.34 | **Pro-231 (O)** | **His-590 (NE2)** | 2.47 |
|  | **Cys-254 (N)** | **Ala-591 (O)** | 2.01 | **Cys-254 (N)** | **Ala-591 (O)** | 2.13 |
|  | **Pro-308 (O)** | **Glu-509 (N)** | 2.07 | **Asp-255 (N)** | **Thr-593 (OG1)** | 2.27 |
|  | Ser-380 (OG) | Thr-478 (OG1) | 2.74 | **Asp-255 (OD1)** | **Thr-593 (OG1)** | 2.67 |
|  | **Thr-398 (OG1)** | **Tyr-481 (N)** | 2.07 | Asn-304 (O) | Lys-513 (NZ) | 2.75 |
|  |  |  |  | His-306 (N) | Glu-509 (OE1) | 2.19 |
|  |  |  |  | Ser-307 (N) | Glu-509 (OE2) | 2.13 |
|  |  |  |  | Pro-308 (O) | Glu-509 (N) | 1.93 |
|  |  |  |  | Asp-355 (OD1) | Gln-436 (NE2) | 3 |
|  |  |  |  | Asp-377(OD1) | Tyr-432 (OH) | 2.68 |
|  |  |  |  | Asp-377 (OD2) | Tyr-432 (OH) | 2.8 |
|  |  |  |  | **Thr-398 (OG1)** | **Tyr-481 (N)** | 2.08 |
| **run2** | Asp-148(N) | Asp-639(OD1) | 1.87 | Ser-145(OG) | Gly-676(N) | 1.76 |
|  | **Cys-254(N)** | **Ala-591(O)** | 1.87 | **Cys-254(N)** | **Ala-591(O)** | 1.88 |
|  | **Asn-204(N)** | **Thr-587(O)** | 2.09 | Asn-304(OD1) | Glu-509(N) | 1.88 |
|  | **Thr-398(OG1)** | **Tyr-481(N)** | 2.14 | Glu-200(O) | Thr-587(N) | 1.9 |
|  | **Pro-308(O)** | **Glu-509(N)** | 2.17 | **Thr-398(OG1)** | **Tyr-481(N)** | 2.07 |
|  | **Asn-95(OD1)** | **Ala-684(N)** | 2.2 | Asp-121(O) | Thr-683(N) | 2.1 |
|  | **Asn-95(O)** | **Thr-683(N)** | 2.21 | Glu-200(N | Asp-638(O) | 2.2 |
|  | Asp-355(OD1) | Tyr-507(OH) | 2.55 | **Pro-231(O)** | **His-590(NE2)** | 2.21 |
|  | Asp-355(OD1) | Tyr-507(OH) | 2.55 | Thr-203(N) | Lys-585(O) | 2.22 |
|  | **Gln-94(O)** | **Thr-683(OG1)** | 2.64 | Thr-199(N) | His-637(ND1) | 2.33 |
|  | Ser-147(OG) | Asp-639(OD2) | 2.75 | Thr-199(OG1) | His-637(ND1) | 2.38 |
|  | Ser-147(OG) | Asp-639(OD2) | 2.75 | **Asn-204(O)** | **His-590(NE2)** | 2.48 |
|  | Ser-380(OG) | Pro-479(O) | 2.77 | Asp-335(OD2) | Tyr-507(OH) | 2.56 |
|  |  |  |  | Ser-380(OG) | Thr-478(OG1) | 2.64 |
|  |  |  |  | Ser-380(OG) | Thr-478(OG1) | 2.64 |
|  |  |  |  | **Asp-255(OD2)** | **Thr-594(OG1)** | 2.74 |
|  |  |  |  | **Asp-255(OD2)** | **Thr-594(OG1)** | 2.74 |
|  |  |  |  | Tyr-310(OH) | Asn-474(ND2) | 2.75 |
|  |  |  |  | Thr-199(OG1) | Asp-636(O) | 2.79 |
|  |  |  |  | Lys-74(NZ) | Tyr-70(O) | 2.83 |
|  |  |  |  | Thr-199(OG1) | His-637(ND1) | 2.88 |
|  |  |  |  | Asp-148(OD2) | Arg-640(NH2) | 2.93 |
|  |  |  |  | Pro-73(O) | Lys-426(N) | 3.1 |
|  |  |  |  | Tyr-72(N) | Asp-71(OD1) | 3.38 |
|  |  |  |  | Leu-401(O) | Tyr-481(O) | 3.46 |
|  |  |  |  |  |  |  |
| ***A.thaliana*** | **Leu-101 (N)** | **Asp-79 (OD1)** | 2.09 | **Arg-82 (NH1)** | **Ile-78 (O)** | 2.77 |
| **run1** | **Ala-103 (O)** | **Thr-707 (N)** | 2.29 | Asp-129 (O) | Thr-707 (N) | 2.12 |
|  | **Asp-156 (OD2)** | **Arg-661 (NE)** | 2.01 | Gly-154 (O) | Gly-704 (N) | 1.98 |
|  | **Thr-212 (O)** | **Thr-604 (N)** | 1.82 | **Gly-209 (O)** | **Asp-659 (N)** | 1.92 |
|  | **Asn-215 (N)** | **Thr-604 (O)** | 1.73 | Thr-212 (O) | Thr-604 (N) | 1.83 |
|  | Asn-215 (O) | His-607 (NE2) | 2.34 | Asn-215 (N) | Thr-604 (O) | 1.99 |
|  | Asp-243 (OD1) | Ser-611 (OG) | 2.65 | **Asn-215 (O)** | **His-607 (NE2)** | 1.67 |
|  | **Pro-325 (O)** | **Glu-526 (N)** | 2.18 | Asp-266 (OD2) | Ser-611 (OG) | 2.57 |
|  | **Ser-395 (OG)** | **Thr-495 (OG1)** | 2.53 | Asp-266 (OD1) | Ser-611 (OG) | 2.4 |
|  | **Ser-395 (OG)** | **Pro-496 (O)** | 2.57 | Asn-321 (ND2) | Glu-526 (OE2) | 2.78 |
|  | Thr-413 (OG1) | Phe-498 (N) | 2.42 | Pro-325 (O) | Glu-526 (N) | 2.46 |
|  |  |  |  | Pro-325 (O) | Glu-526 (OE2) | 2.15 |
|  |  |  |  | **Asp-370 (OD2)** | **Tyr-524 (OH)** | 2.77 |
|  |  |  |  | **Asp-370 (OD2)** | **Tyr-524 (N)** | 2.70 |
|  |  |  |  | Ser-395 (OG) | Pro-496 (O) | 2.56 |
|  |  |  |  | **Thr-413 (OG1)** | **Phe-498 (N)** | 2.41 |
| **run2** | **Asn-215(N)** | **Thr-604(O)** | 1.79 | Leu-101(N) | Asp-79(OD2) | 1.82 |
|  | **Asp-156(OD2)** | **Arg-661(NE)** | 1.92 | Ser-133(OG) | Gly-704(N) | 2.07 |
|  | Asp-156(N) | Asp-660(OD1) | 1.98 | Asn-215(N) | Thr-604(O) | 2.21 |
|  | **Ala-103(O)** | **Thr-707(N)** | 2.02 | Tyr-197(OH) | His-607(NE2) | 2.27 |
|  | **Pro-325(O)** | **Glu-526(N)** | 2.04 | **Gly-209(O)** | **Asp-659(N)** | 2.31 |
|  | **Leu-101(N)** | **Asp-79(OD1)** | 2.05 | **Thr-413(OG1)** | **Phe-498(N)** | 2.33 |
|  | Asn-215(O) | His-607(NE2) | 2.07 | Ser-214(N) | Lys-602(O) | 2.34 |
|  | **Thr-212(O)** | **Thr-604(N)** | 2.12 | Ser-414(O) | Phe-498(N) | 2.36 |
|  | Trp-157(N) | Asp-660(OD2) | 2.4 | Tyr-327(OH) | Glu-526(N) | 2.4 |
|  | Gln-102(O) | Thr-707(OG1) | 2.59 | Val-441(O) | Gly-443(N) | 2.41 |
|  | Ser-155(OG) | Asp-660(OD2) | 2.66 | **Asn-215(O)** | **His-607(NE2)** | 2.49 |
|  | Ser-155(OG) | Asp-660(OD2) | 2.66 | Gln-102(O) | Thr-707(OG1) | 2.51 |
|  | Asp-266(OD1) | Ser-611(OG) | 2.67 | Asp-266(OD1) | Ser-491(OG) | 2.6 |
|  | **Ser-395(OG)** | **Pro-496(O)** | 2.67 | Asp-266(OD1) | Ser-491(OG) | 2.6 |
|  | **Ser-395(OG)** | **Thr-495(OG1)** | 2.73 | **Asp-370(OD1)** | **Tyr-524(OH)** | 2.61 |
|  | Tyr-80(N) | Ile-78(O) 2.7 | 2.76 | **Asp-370(OD1)** | **Tyr-524(OH)** | 2.61 |
|  |  |  |  | **Arg-82(NH1)** | **Ile-78(O)** | 2.79 |
